# Danggui Shaoyao San attenuates depressive-like behaviors in mice via TLR4/NF-κB p65/JAK-STAT3/AKT-GSK3β signaling pathways: modulation of hippocampal neurogenesis and neuroinflammation

**DOI:** 10.3389/fnut.2025.1652968

**Published:** 2025-10-20

**Authors:** Chuan-Feng Tang, Fan Li, Lu-Han Ma, Qiao-Na Wang, Peng-Fei Xie, Lang Xiang, Yu-Jie Zhu, Yue-Yao Wang, Yi-Zhu Zhang, Jun-Jie Shi, Sheng-Jie Li, Jian-Mei Li

**Affiliations:** 1 State Key Laboratory of Technologies for Chinese Medicine Pharmaceutical Process Control and Intelligent Manufacture, Department of Pharmacy, Nanjing University of Chinese Medicine, Nanjing, China; 2 School of Food Science, Nanjing Xiaozhuang University, Nanjing, China; 3 School of Food Science and Pharmaceutical Engineering, Nanjing Normal University, Nanjing, China

**Keywords:** DSS, depression, hippocampal neurogenesis, hippocampal inflammation, TLR4/NF-κB p65, JAK2/STAT3 and AKT-GSK3β signaling pathways

## Abstract

**Purpose:**

Danggui Shaoyao San (DSS), a traditional Chinese herbal formula abundantly containing both medicinal and dietary components, was first documented in Jin Gui Yao Lue (Synopsis of the Golden Chamber) by Zhang Zhongjing during the Eastern Han Dynasty of China. Depression, a multifactorially induced affective disorder, has its precise etiological factors and underlying pathophysiological mechanisms remaining incompletely understood. Therefore, this study aimed to investigate the therapeutic effects of DSS on corticosterone-induced depression in mice and clarify the underlying mechanisms.

**Methods:**

The therapeutic effects of DSS on depressive-like behaviors were assessed via behavioral tests in mice. Potential therapeutic targets of DSS were explored via network pharmacology and RNA sequencing (RNA-seq) approaches. Furthermore, immunofluorescence staining was utilized to evaluate neuroinflammatory responses and hippocampal neurogenesis. Additionally, Western blot analysis was performed to verify the molecular mechanisms underlying DSS-mediated alleviation of depressive-like behaviors in mice.

**Results:**

Network pharmacology analysis revealed 12 phytochemical constituents targeting 168 depression-associated genes. RNA-seq and immunofluorescence staining analyses demonstrated that DSS attenuates hippocampal neuroinflammation via suppressing microglial activation and enhances hippocampal neurogenesis by restoring the number of neural stem cells (NSCs). At the mechanistic level, DSS mitigates depressive-like behaviors in mice through coordinated modulation of the TLR4/NF-κB p65, JAK2/STAT3, and AKT-GSK3β signaling pathways. Collectively, these results position DSS as a potential adjuvant intervention that concurrently modulates both neuroinflammatory responses and hippocampal neurogenesis in depression.

**Conclusion:**

Collectively, our study deciphers the anti-depressant mechanisms of DSS via TLR4/NF-κB p65, JAK2/STAT3 and AKT-GSK3β signaling pathways, while establishing a drug development paradigm that bridges ethnopharmacology and modern systems biology. This integration offers a blueprint for exploiting traditional medicines in CNS drug discovery.

## 1 Introduction

Depression is a heterogeneous neuropsychiatric condition characterized by persistent low mood, anhedonia, and sleep disturbances (1, 2). Global epidemiological data from the World Health Organization (WHO) indicates that over 5% of the global adult population experiences depression, a condition projected to emerge as the leading cause of disability by 2030 (3, 4). Its etiology arises from intricate interactions among genetic predisposition, psychological stressors, biochemical imbalances, and environmental factors. Current antidepressant therapies are associated with significant limitations, including delayed onset of therapeutic effect, elevated suicide risk, and poor patient compliance due to adverse reactions (e.g., fluoxetine-induced reproductive toxicity) (5). T Traditional Chinese Medicine (TCM), characterized by its multi-component and multi-target therapeutic approach, provides a promising paradigm to address these challenges. Classical TCM texts classify depressive syndromes into “Yuzheng” (depressive disorders) and “Baihebing” (lily disease), with treatment principles documented as early as the Han Dynasty (6).

Notably, emerging evidence demonstrates that dietary interventions effectively regulate depressive symptoms. The Mediterranean diet, a pattern rich in polyphenol-rich foods, is associated with a significantly reduced risk of depression (7, 8). This “nutrition-brain axis” mechanism indicates that functional foods with dual medicinal and dietary properties may provide novel preventive and therapeutic strategies for depression. Danggui Shaoyao San (DSS), a classical traditional Chinese medicine (TCM) formula, was first documented in Jin Gui Yao Lue (Synopsis of the Golden Chamber) by Zhang Zhongjing during the Eastern Han Dynasty of China. It consists of multiple herbal ingredients, including *Angelica sinensis* (Oliv.) *Diels* (Danggui), *Paeonia lactiflora* (Shaoyao), *Atractylodes macrocephala Koidz*. (Baizhu), *Poria cocos* (Schw.) *Wolf* (Fuling), *Alisma orientale* (sam.) juzep (Zexie), and *Ligusticum chuanxiong Hot* (Chuanxiong). Notably, *Poria cocos* and *Atractylodes macrocephala* possess dual medicinal-food applications, this establishes a key rationale for the comparative advantage of DSS over conventional antidepressants in food-drug safety. Conventional antidepressants are frequently accompanied by significant adverse effects, poor patient adherence, and uncertain long-term risks. In contrast, DSS, a classical TCM formula with a long-standing history of safe clinical use, mitigates concerns regarding potential toxic effects associated with synthetic pharmaceutical agents. DSS is traditionally used to alleviate menstrual pain and other abdominal pain types in women, and recent studies have elucidated its antioxidant, antithrombotic, cognitive, and neuroprotective properties (9, 10). The multi-target regulatory effects of this traditional Chinese herbal formula represent another key comparative advantage of DSS over conventional antidepressant therapies. Most conventional antidepressant therapies primarily target a single or limited number of neurotransmitter systems (e.g., monoamine reuptake inhibition), leading to incomplete symptom relief or suboptimal efficacy in some patients. In contrast, DSS modulates multiple pathways and molecular targets, which aligns with the complex multifactorial pathogenesis of depression, thus holding promise for more comprehensive therapeutic outcomes. Studies have demonstrated that it activates the hippocampal dopaminergic system and enhances acetylcholine synthesis, thereby improving memory-related behaviors and learning capabilities (11–13). Based on these findings, we hypothesize that DSS may potentially alleviate depression.

As scientific research advances, the underlying pathogenesis of depression is progressively being elucidated. Accumulating evidence indicates that depression is closely associated with pathological mechanisms including neuroinflammation, impaired neurogenesis, and metabolic dysregulation (14). Neuroinflammation, driven by microglial overactivation, disrupts serotonergic/dopaminergic neurotransmitter systems and impairs hippocampal neurogenesis—a process contingent on neural stem cell (NSC) proliferation and differentiation within the hippocampal dentate gyrus and subventricular zone (SVZ) (14–18). Activation of key signaling pathways (e.g., TLR4-NF/κB p65 and JAK2/STAT3) not only exacerbates neuroinflammation but also exacerbates depressive-like behaviors by suppressing hippocampal neurogenesis (19, 20). The AKT-GSK3β signaling pathway is pivotal in regulating neural cell proliferation and differentiation; dysregulation of this pathway directly impairs neuronal survival and synaptic plasticity (21).

Given the multifactorial pathogenesis of depression and the limitations of current antidepressant therapies, developing safe and effective treatment strategies remains a key research priority. TCM formulas, defined by their multi-component and multi-target pharmacological characteristics, exhibit considerable therapeutic potential for depression. In this study, we utilized RNA-seq combined with network pharmacology to elucidate the mechanistic pathways by which DSS alleviates depressive symptoms. Our findings reveal that DSS mitigates hippocampal neuroinflammation and enhances hippocampal neurogenesis in depression model mice via modulation of the TLR4/NF-κB p65, JAK2/STAT3 and AKT-GSK3β signaling pathways. Additionally, DSS modulates metabolic processes of amino acids and lipids while concurrently ameliorating depressive-like behaviors in these model systems.

## 2 Materials and methods

### 2.1 Herbs

According to a previous study, *Angelica sinensis* (Oliv.) Diels (Danggui) (Cat#259230520), *Paeonia lactiflora* (Shaoyao) (Cat#223230201), *Atractylodes macrocephala Koidz*. (Baizhu) (Cat#224230204), *Poria cocos* (Schw.) Wolf (Fuling) (Cat#220501), *Alisma orientale* (sam.) juzep (Zexie) (Cat#220601), and *Ligusticum chuanxiong Hot*. (Chuanxiong) (Cat#220601) were bought from Guoxin Pharmacy, Nanjing, China (22).

### 2.2 Animals

Male C57BL/6J mice (6-week-old) were purchased from GemPharmatech Co. Animal husbandry and experimental procedures were approved by the Animal Ethics and Welfare Committee of Nanjing University of Traditional Chinese Medicine (Approval No. 202406A042).

### 2.3 Construction of corticosterone depression mouse model

After 2 weeks of acclimatization, the mice were randomly assigned into control group (CTL), model group (Corticosterone, cort), DSS low-dose treatment group (DSS-L group, 0.428571 g/kg, clinically equivalent dose), DSS high-dose treatment group (DSS-H group, 1.714286 g/kg), and positive drug fluoxetine group (10 mg/kg/day, administered by gavage). Saline was given to the control group; in the model group, corticosterone 20 mg/kg was given subcutaneously for 3 weeks in the DSS treatment group and the positive drug fluoxetine group. Model validity was assessed using depressive-like behavioral score. Drug administration began 1 week after the initiation of corticosterone-induced modeling. Behavioral tests were conducted following 2 weeks of treatment, and the results were used to evaluate the therapeutic efficacy.

### 2.4 Behavioral testing

**Open field testing (OFT):** The OFT was performed using an empty box of 25 cm × 25 cm × 40 cm, and the field was divided into nine identical squares. Each mouse was placed individually at the center of the site and allowed to explore freely for 10 min. Using Visu Track, we recorded the time and distance the mouse spent around the perimeter and at the center of the site, as well as its total distance traveled. The mice were removed after each test and the equipment was cleaned using 70% ethanol to remove any odor signals.

**Sucrose preference test (SPT):** Each mouse was separated into a cage for 3 days and given a bottle of plain water and 1% sucrose solution to acclimatize for 2 days, the position of the bottle was changed every 24 h during acclimatization. At the end of acclimatization, mice were abstained from water for 24 h and then re-offered a bottle of plain water and a bottle of 1% sucrose solution for the next 2 h (8:00–10:00 PM). The total weight of each bottle was measured before and after the test to obtain the weight of the liquid consumed. Sugar preference was calculated as [Sucrose water intake / (Sucrose water intake+Purified water intake)] × 100%.

**Forced swimming test (FST):** Mice were acclimatized to the swimming environment for 5 min the day before the official FST. The next day, mice were placed individually in a 15 cm deep glass cylinder (20 cm diameter × 50 cm height) at 24 ± 1 °C for 5 min. The time spent floating, swimming, and struggling was recorded using Visu Track.

**Tail suspension test (TST):** Mice were isolated in a separate room with the end of their tails secured to a suspension device approximately 50 cm above the floor for 6 min. The time the mice spent struggling, climbing and not moving was recorded using Visu Track.

### 2.5 RNA sequence (RNA-seq)

Hippocampal tissue samples were collected from mice assigned to three groups: the control group (CTL), corticosterone-induced model group (CORT), and DSS-treated group (DSS), with 5 mice per group (*n* = 5/group). All tissue samples were sent to Oebiotech Co., Ltd. (Beijing, China) for subsequent RNA sequencing and data analysis. For the identification of differentially expressed genes (DEGs), the screening thresholds were set as follows: absolute log_2_ fold change (|log_2_FC|) ≥ 2.0 and raw *p*-value ≤ 0.05. Functional annotation of DEGs was conducted via Gene Ontology (GO) enrichment analysis and Kyoto Encyclopedia of Genes and Genomes (KEGG) pathway enrichment analysis. Enriched GO terms and KEGG pathways with a corrected *p*-value (false discovery rate, FDR) < 0.05 were considered statistically significant. To visualize the expression patterns of differentially expressed mRNAs, hierarchical clustering analysis was performed using their Fragments Per Kilobase of transcript per Million mapped reads (FPKM) values (after log_2_ transformation and quantile normalization), and the clustering results were presented as a heatmap.

### 2.6 Network pharmacology

Identification of active ingredients and prediction of active ingredient targets of DSS: Using the Traditional Chinese Medicine Systematic Pharmacology Database and Analysis Platform (TCMSP).^1^ The active ingredients of DSS were screened. The screening conditions were set as bioavailability (OB) ≥ 30% and drug-like properties (DL) ≥ 0.18 to obtain the active ingredients of each herb. The DrugBank^2^ and SwissTargetPrediction^3^ databases were used to obtain the target of action of each active ingredient.

Identification of potential targets for the treatment of depression by DSS: The Therapeutic Target Database (TTD)^4^ and drugbank^2^ databases were used to obtain depression disease targets. Using Venny database,^5^ there were two sets of data, one was the target genes obtained from DSS powder, and the other was the target genes related to depression. The target genes obtained from DSS and those related to depression were screened for overlap. The intersecting genes were obtained and regarded as potential targets of DSS for depression.

Constructing networks of drugs, components, and targets (including protein-protein interactions): The STRING^6^ database is used to predict protein-protein interaction (PPI) networks. Use Cytoscape 3.9.0 software to build network models of drugs, components, targets and diseases.

Enrichment analysis: To determine the biological functions and signaling pathways involved in the common targets of the drug active ingredients and depression, Gene Ontology (GO) and Kyoto Encyclopedia of Genes and Genomes (KEGG) pathway enrichment analyses were performed using the DAVID.^7^ *P*-values ≤ 0.05 for GO and the KEGG pathway were considered significantly enriched.

Molecular docking: Molecular docking software Autodock vina was selected for the molecular docking process in this study. The key active ingredients of DSS were found in the TCMSP database in mol2 format. The core target structure was obtained from the PDB database.^8^ The research objects were the active ingredients and core targets of DSS. Molecular docking of the active ingredients and core targets of DSS was carried out using AutoDock vina software. The results obtained from the molecular docking were visualized and analyzed using PyMOL software.

### 2.7 Immunofluorescence staining

After anesthetizing the mice with pentobarbital, the hearts were perfused with saline and 4% PFA. Whole brains were removed, fixed in 4% PFA overnight, transferred to 0.4% PFA and stored at 4 °C. The brains were then embedded in optimal cutting temperature compound (4583, Sakura Finetek, Torrance, CA, USA) and sliced into 30 μm-thick coronal sections using a cryosectioner (Leica, Wetzlar, Germany). Immunostaining was performed on hippocampal sections. Sections were washed three times with phosphate buffer saline (PBS) for 5 min each time, incubated with QuickBlock™ Blocking Buffer containing 0.1% Triton™ X-100 for 1 h. Sections were incubated with primary antibody overnight at 4 °C, and then incubated with fluorescently labeled secondary antibody at room temperature for 2 h in the dark. After incubation for 2 h in the dark, the sections were washed three times with phosphate buffer saline (PBS). Sections were blocked with anti-fluorescent burst sealer and images were captured using a Leica TCS SP8 confocal microscope. Immunofluorescence results were quantified using ImageJ software. Primary antibodies used were Rabbit anti-Iba-1 (1:500, Wako, 019-19741), Rabbit anti-GFAP (1:500, Invitrogen, PA5-16291), Rabbit anti-DCX (1:500, Abcam, ab104224), Mouse anti-Nestin (1:500, Abcam, ab6142), Rabbit anti-NeuN (1:500, Abcam, ab177487); secondary antibodies used were Goat anti-Rabbit IgG (H+L) Cross-Adsorbed Secondary Antibody, Alexa Fluor™ 488 (1:500, Invitrogen, A-11008), Goat anti-Mouse IgG (H+L) Cross-Adsorbed Secondary Antibody, Alexa Fluor™ 488 (1:500, Invitrogen, A-11001), Goat anti-Rat IgG (H+L) Cross-Adsorbed Secondary Antibody, Alexa Fluor™ 555 (1:500, Invitrogen, A-21434), Goat anti-Mouse IgG (H+L) Cross-Adsorbed Secondary Antibody, Alexa Fluor™ 555 (1:500, Invitrogen, A-21422).

### 2.8 Western blotting

Mouse hippocampal tissue was homogenized in ice-cold RIPA buffer containing protease inhibitors and phosphatase inhibitors. Samples were then centrifuged at 12,000 rpm for 15 min at 4 °C to obtain supernatant protein lysates. Protein concentration was assayed using a bicinchoninic acid (BCA) protein concentration assay kit using bovine serum albumin as a standard. Aliquots of protein samples were separated by sodium dodecyl sulfate-polyacrylamide gel electrophoresis and subsequently transferred to a polyvinylidene difluoride membrane (IPVH00010, Millipore). The membranes were closed with protein-free rapid closure solution (G2052-500 ML, Servicebio) for 5 min at 4 °C with anti-Mouse anti-GAPDH (Cell signaling, 97166, 1: 1000), Mouse anti-β-Actin (Cell signaling, 3700, 1:1000), Rabbit anti-p-stat3 (Cell signaling, 9145, 1:1000), Rabbit anti-stat3 (Cell signaling, 30835, 1:1000), Rabbit anti-NF-κB p65 (Cell signaling, 8242, 1:1000), Rabbit anti-p-NF-κB p65 (Cell signaling, 3033, 1:1000), Rabbit anti-p-AKT (Cell signaling, 9271, 1:1000), Rabbit anti-AKT (Cell signaling, 9272, 1:1000), Rabbit anti-p-JAK2 (Affinity, AF3024, 1:1000), Rabbit anti-JAK2 (Affinity, AF6022, 1:1000), Rabbit anti-TLR4 (Cell signaling, 82658, 1:1000), Rabbit anti-p-GSK3β (Cell signaling, 9336, 1:1000), and Rabbit anti-GSK3β (Cell signaling, 12456, 1:1000) were incubated overnight with primary antibody shaking. Afterward, the membranes were incubated with secondary antibody HRP-conjugated Goat Anti-Rabbit IgG(H+L) (Wuhan Sanying, SA00001-2, 1:5000) or HRP-conjugated Goat Anti-Mouse IgG (Wuhan Sanying, SA00001-1-A, 1:5000) for 1 h at room temperature. The grayscale values of the bands were quantified using the ImageJ software. The grayscale values of the bands were quantified using the ImageJ software.

### 2.9 Statistics

All data was presented as mean ± standard error of mean (SEM). Two-tailed Student’s *t*-test was carried out for two group comparisons. Data of three and more groups with single factor variance, were analyzed by one-way ANOVA for comparisons. *P* < 0.05 is statistically significant.

## 3 Results

### 3.1 Corticosterone induces depressive-like behaviors mainly through impairing the hippocampal neurogenesis and promoting neuroinflammation in mice

**FIGURE 1:**
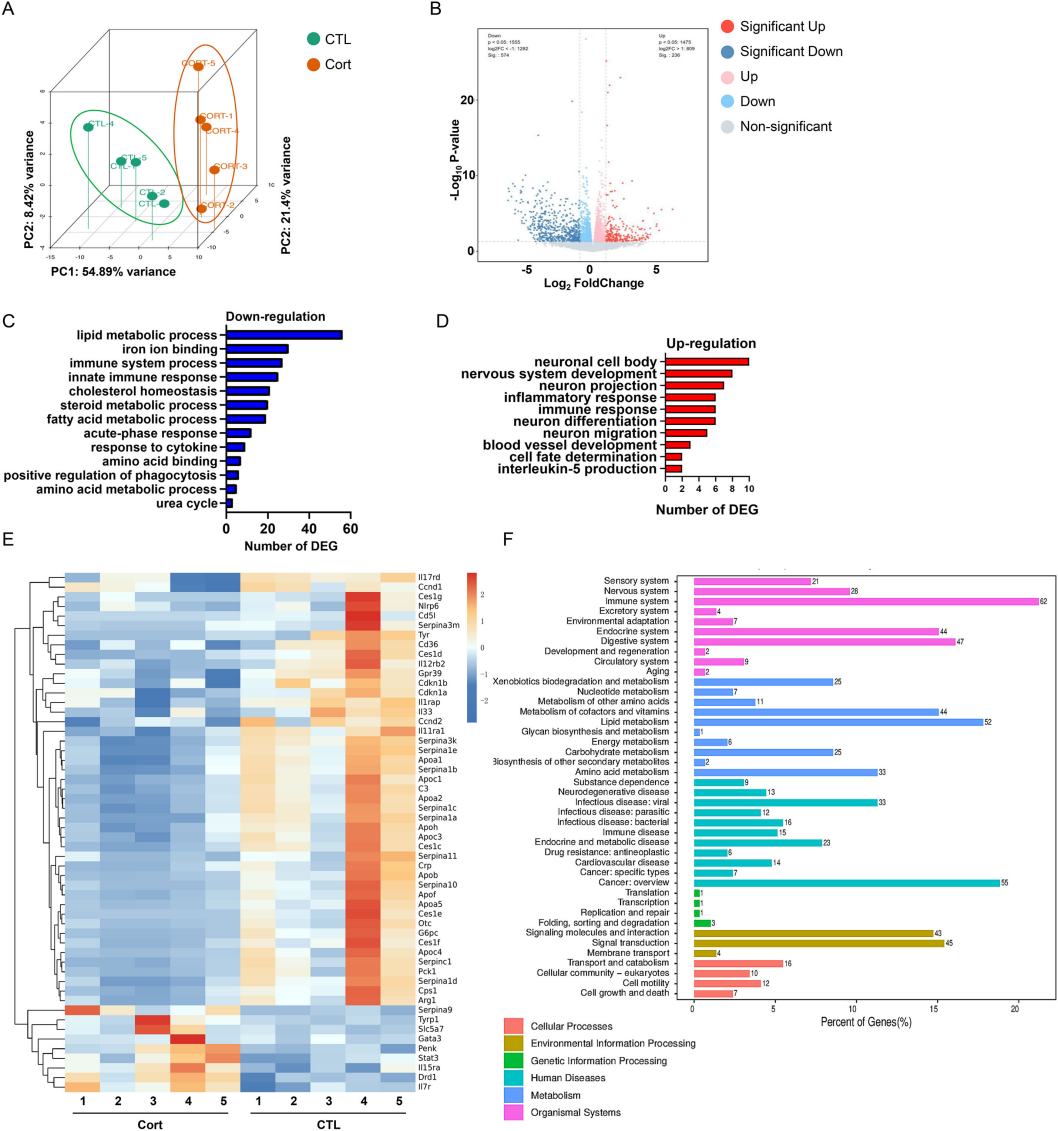
Effect of corticosterone treatment on gene expression profiles in mouse hippocampus. **(A)** Principal component analysis (PCA) of the RNA sequencing data showed significant clustering among groups. **(B)** Volcano plot of differentially expressed genes in the corticosterone-treated group (Cort) and control group (CTL). **(C)** GO enrichment analysis of down-regulated genes was used to clarify the biological function categories in which the down-regulated genes were mainly involved. **(D)** GO enrichment analysis of up-regulated genes. **(E)** Heatmap showing transcriptional changes in corticosterone-treated and control mice. Significant associations of transcriptional changes with hippocampal metabolism, inflammation and neurogenesis are reflected. **(F)** Visualization of the KEGG pathway enrichment analysis of 816 differential genes.

Corticosterone was employed to induce depressive-like behaviors in mice. Principal component analysis (PCA) plot of hippocampal RNA sequencing data demonstrated distinct clustering the corticosterone-treated mice and the control mice (Figure 1A). Differential gene analysis revealed 239 upregulated and 577 downregulated genes in corticosterone-treated versus control mice (Figure 1B). Gene ontology (GO) enrichment analysis was separately applied to upregulated and downregulated genes to identify biological processes and molecular functions associated with depression pathogenesis. GO analysis of upregulated genes revealed enrichment in biological processes including nervous system development, neuron projection, neuron differentiation, cell fate determination, immune response, and inflammatory response (Figure 1C). Downregulated genes exhibited significant enrichment in biological processes such as immune system processes, cytokine response, innate immune response, positive regulation of phagocytosis, lipid metabolic processes, amino acid metabolic processes, acute-phase response, steroid metabolic processes, and fatty acid metabolic processes (Figure 1D). Additionally, transcriptomic changes between corticosterone-treated and control mice, visualized in the heatmap, were significantly associated with hippocampal metabolism, inflammation, and neurogenesis (Figure 1E). Genes involved in cell cycle, inflammation, and phagocytic function included Ccnd2, Cdkn1a, Cdkn1b, Nlrp6, Il4ra, Il5ra, Il33, Cd36, C3, et al. (Figure 1E), which regulate NSC proliferation, neurogenesis, and microglia-mediated hippocampal inflammation (23–27). Additionally, KEGG pathway enrichment analysis was conducted for these 816 differentially expressed genes. The results indicated that these genes were primarily involved in pathways such as immune response (62 pathways), energy metabolism (6 pathways), and cell growth and death (7 pathways) (Figure 1F). In conclusion, these findings suggest that corticosterone may induce depression in mice by impairing hippocampal neurogenesis and promoting neuroinflammatory responses.

### 3.2 DSS and fluoxetine improves corticosterone induced depressive-like behaviors of mice

**FIGURE 2:**
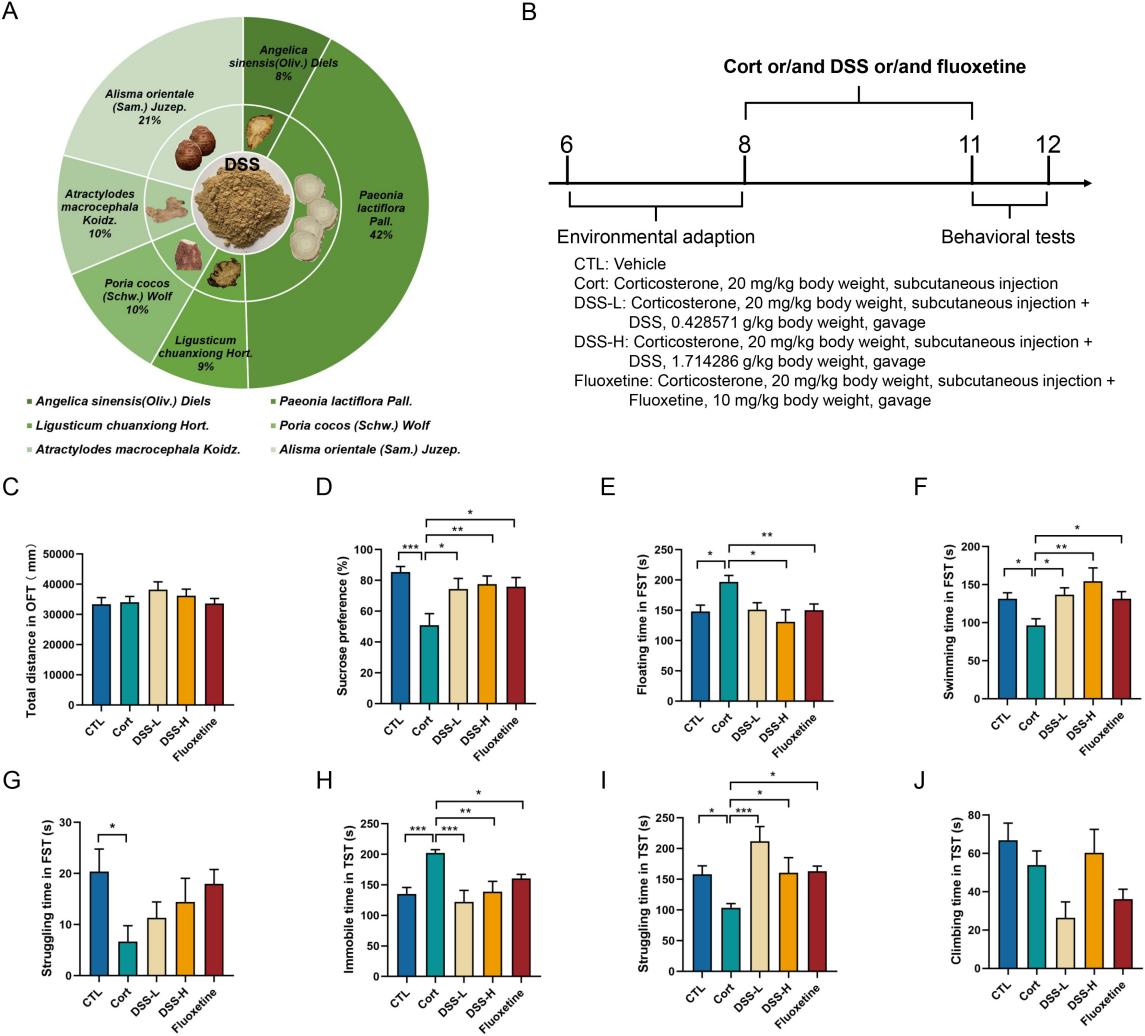
Effect of DSS on corticosterone-induced depressed mice. **(A)** The proportion of each component in DSS. **(B)** Experimental timeline of corticosterone and DSS intervention and behavioral tests. **(C)** Statistical results of the total distance in OFT. **(D)** Statistical results of the sucrose preference. **(E)** Statistical results of the floating time in FST. **(F)** Statistical results of the swimming time in FST. **(G)** Statistical results of the struggling time in FST. **(H)** Statistical results of the immobile time in TST. **(I)** Statistical results of the struggling time in TST. **(J)** Statistical results of the climbing time in TST. Data are expressed as Mean ± Sem, *n* ≥ 9, *P < 0.05, **P < 0.01, ***P < 0.001.

The composition of DSS, including the proportion of each component, is presented in Figure 2A. During the induction of a depression model with corticosterone, low- and high-dose DSS were, respectively, administered via gavage, and fluoxetine was chosen as a positive control antidepressant (Figure 2B). Corticosterone successfully induced depressive-like behaviors in mice. It did not significantly affect total locomotor distance or speed in the open-field test (OFT), but markedly increased immobility time in both the forced swim test (FST) and tail suspension test (TST) (Supplementary Figure 1). Neither DSS nor fluoxetine treatment induced dyskinesia in the open-field test (OFT) (Figure 2C). Both DSS and fluoxetine ameliorated corticosterone-induced anhedonia, as evidenced by a significant increase in sucrose preference relative to corticosterone-induced depressed mice (Figure 2D). DSS and fluoxetine significantly prolonged swimming time and reduced floating time in the forced swim test (FST) in corticosterone-induced depressed mice (Figures 2E–G). Furthermore, DSS and fluoxetine significantly decreased immobility time and increased struggling time in the tail suspension test (TST), indicating that they effectively alleviated behavioral despair in these mice (Figures 2H–J). Collectively, these results indicate that DSS effectively ameliorates both anhedonia and behavioral despair in corticosterone-induced depressive mice.

### 3.3 Screening of active components and target analysis of DSS for depression treatment

The bioactive components of DSS were identified from the TCMSP database based on the following criteria: oral bioavailability (OB) ≥ 30% and drug-likeness (DL) ≥ 0.18. This screening process identified the bioactive components of DSS (Supplementary Table 1), with 12 key components showing significant association with depression (Supplementary Table 2). These components include Mandenol, Myricanone, and Wallichilide from *Ligusticum chuanxiong*; Alisol B monoacetate from *Alisma orientalis*; Stigmasterol from *Angelica sinensis*; Paeoniflorgenone, β-sitosterol, and Kaempferol from *Paeonia lactiflora*; 3β-acetoxyatractylone and 8β-ethoxy atractylenolide from *Atractylodes macrocephala*; and hederagenin from *Poria cocos* (Supplementary Table 2).

**FIGURE 3:**
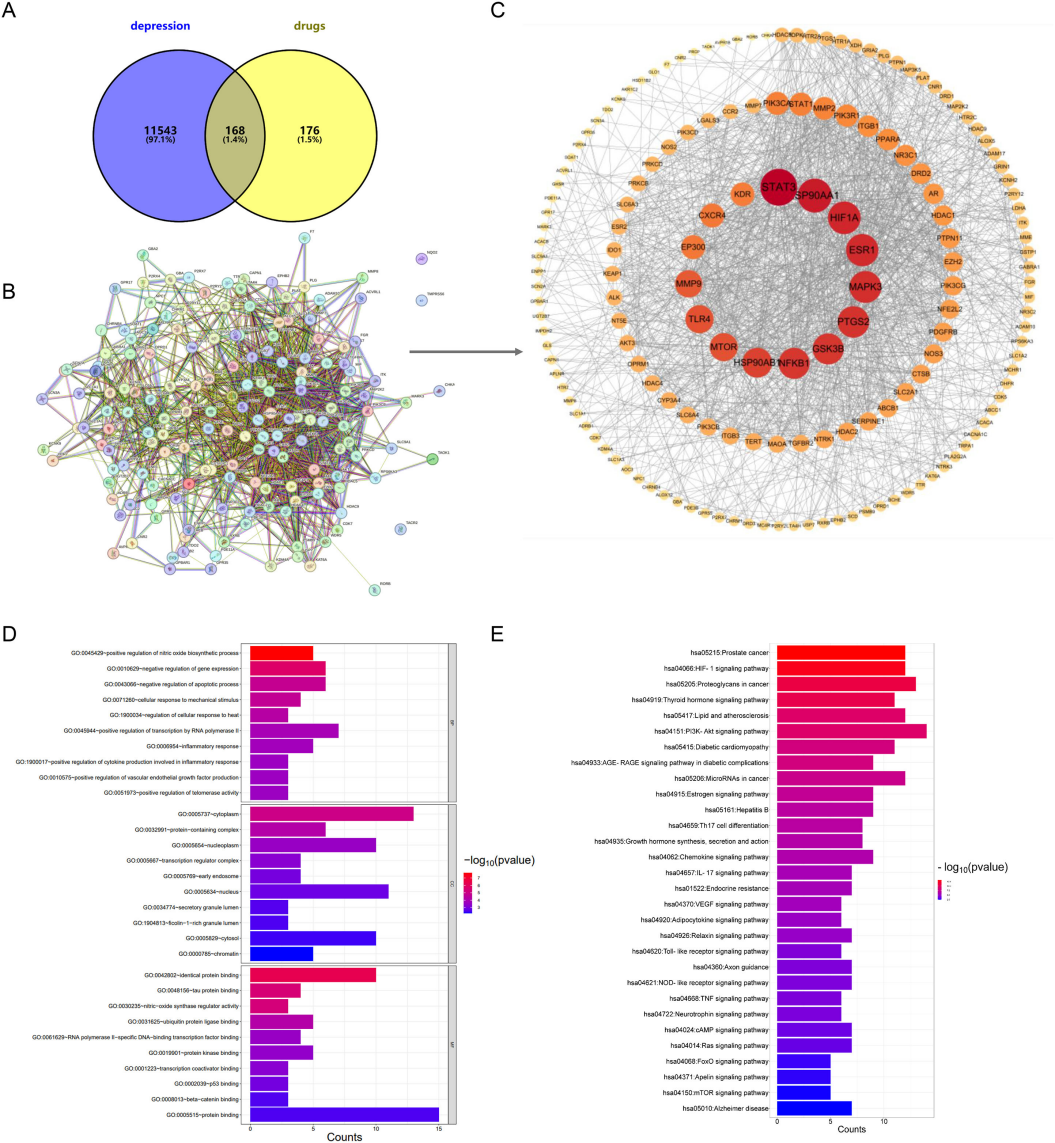
Network pharmacology analyze results. **(A)** Venn diagram of DSS–depression targets. **(B)** PPI network of the common target genes. **(C)** PPI network of the key target genes. **(D)** Visualization of the GO term enrichment analysis for the key targets of DSS in the treatment of depression. **(E)** Visualization of KEGG pathway enrichment analysis.

To investigate the therapeutic mechanisms of DSS in depression, 11,711 depression-associated targets were retrieved from the DrugBank and TTD databases. Of these, 344 targets showed overlap with the 12 bioactive components of DSS (Supplementary Tables 3–15; Supplementary Figure 2). A Venn diagram was generated to visualize the intersection of DSS-associated targets and depression-associated targets, identifying 168 overlapping targets (Figure 3A). These overlapping targets were considered potential therapeutic targets of DSS for depression. These overlapping targets were further analyzed via the STRING database, and a protein-protein interaction (PPI) network was constructed using Cytoscape 3.9.0 (Figure 3B; Supplementary Figure 2A). The PPI network was sorted based on node degree, and the top 15 hub targets with the highest degrees were identified as STAT3, HSP90AA1, ESR1, HIF1A, MAPK3, PTGS2, NFKB1, GSK3β, HSP90AB1, TLR4, MMP9, MTOR, EP300, CXCR4, and KDR (Figure 3C). These hub targets are likely to play key roles in mediating the antidepressant effects of DSS.

GO enrichment analysis of the key DSS targets involved in depression was comprehensively performed. The results revealed significant enrichment of genes associated with the biological process “inflammatory response” (Figure 3D). Notably, “tau protein binding” was also identified as a molecular function. Tau protein, which is closely associated with neuronal function, may contribute to impaired neural activity when its binding is abnormal (Figure 3D). Furthermore, KEGG pathway enrichment analysis identified several signaling pathways potentially involved in mediating the antidepressant effects of DSS. These include pathways regulating cell proliferation and apoptosis (e.g., PI3K-AKT and mTOR signaling pathways) and pathways modulating inflammatory responses (e.g., Toll-like receptor and NOD-like receptor signaling pathways) (Figure 3E). Collectively, these findings suggest that DSS may alleviate corticosterone-induced depression by promoting NSC-mediated neurogenesis and reducing hippocampal neuroinflammation.

### 3.4 Molecular docking analysis reveals key antidepressant mechanisms of DSS via TLR4, STAT3 and AKT signaling pathways

**FIGURE 4:**
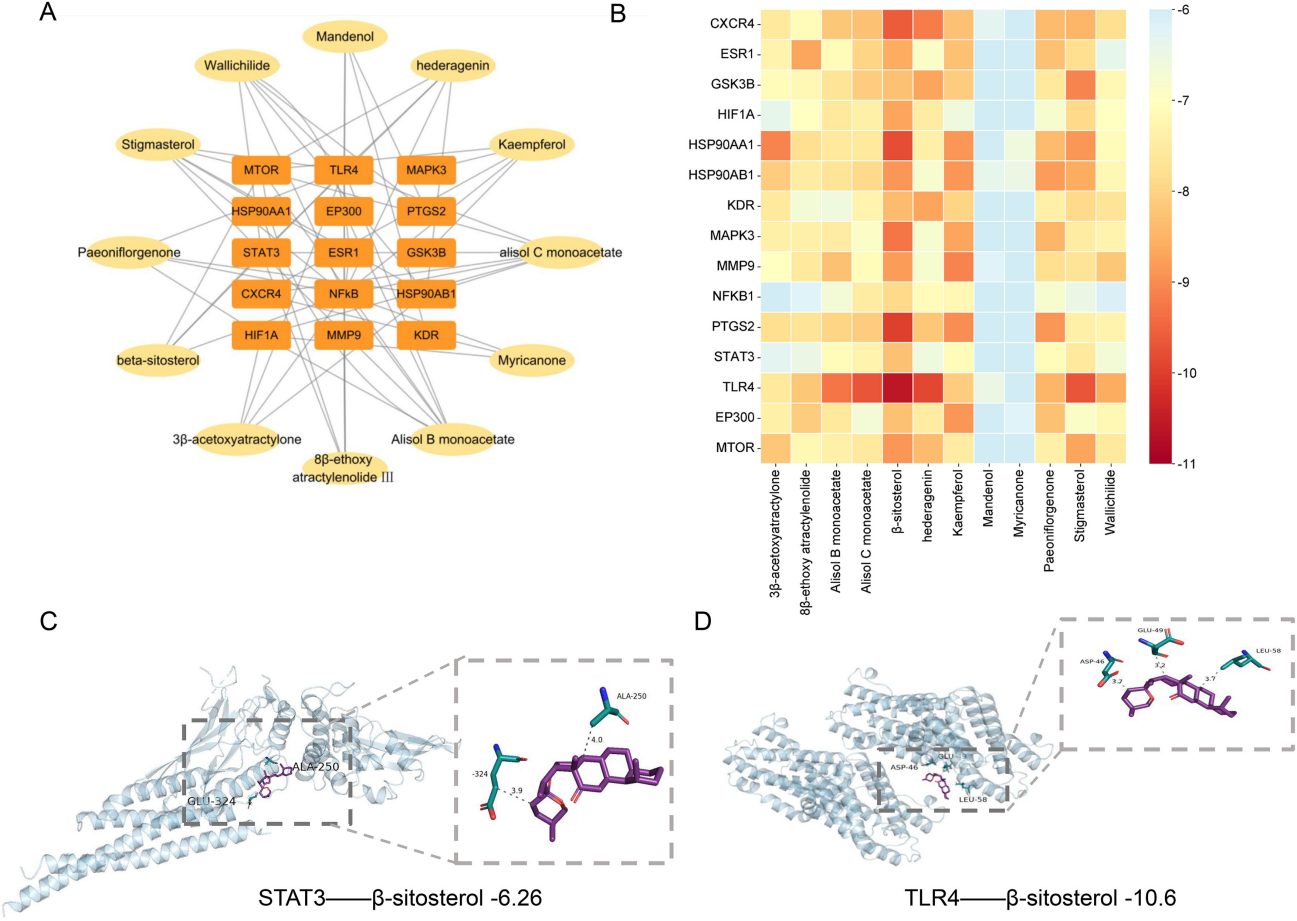
**(A)** Compound-Gene network. The orange rectangles represent key genes, and the yellow ellipses represent active compounds. **(B)** The heatmap of the target molecule docking results of the candidate core active components. **(C–D)** Molecular docking results of the important compounds and the corresponding proteins of the gene targets.

To further elucidate the interactions between the 12 bioactive components of DSS and the 15 hub targets, molecular docking analyses were conducted to predict binding modes and energy values (Figure 4A). Results revealed that β-sitosterol was among the most promising compounds exhibiting potential antidepressant activity, as it displayed the lowest binding free energy with TLR4 (Figure 4B). Among the 12 compounds, Mandenol and Myricanone exhibited superior binding affinities, whereas the remaining 10 compounds also displayed favorable binding free energies with depression-associated targets (Supplementary Table 16). These interactions likely target key proteins in the JAK-STAT3, AKT-GSK3β, and TLR4/NF-κB p65 signaling pathways (Figure 4B), though further experimental validation is warranted. Additionally, 3D binding models were constructed to visualize these interactions. β-sitosterol displayed binding free energies of −6.26 kcal/mol for STAT3 (Figure 4C), −10.6 kcal/mol for TLR4 (Figure 4D), −6.7 kcal/mol for NF-κB p65 (Supplementary Figure 3A), and −9.8 kcal/mol for HSP90AA1 (Supplementary Figure 3B). Similarly, 3β-acetoxyatractylone displayed a binding free energy of −9.1 kcal/mol for HSP90AA1 (Supplementary Figure 3C), and Stigmasterol exhibited a binding free energy of −9.7 kcal/mol for TLR4 (Supplementary Figure 3D). Collectively, these findings suggest that β-sitosterol and other bioactive components may mediate antidepressant effects by modulating key targets within inflammatory and neuroprotective pathways.

### 3.5 DSS ameliorates depressive-like behaviors in mice by enhancing hippocampal neurogenesis and suppressing neuroinflammation

**FIGURE 5:**
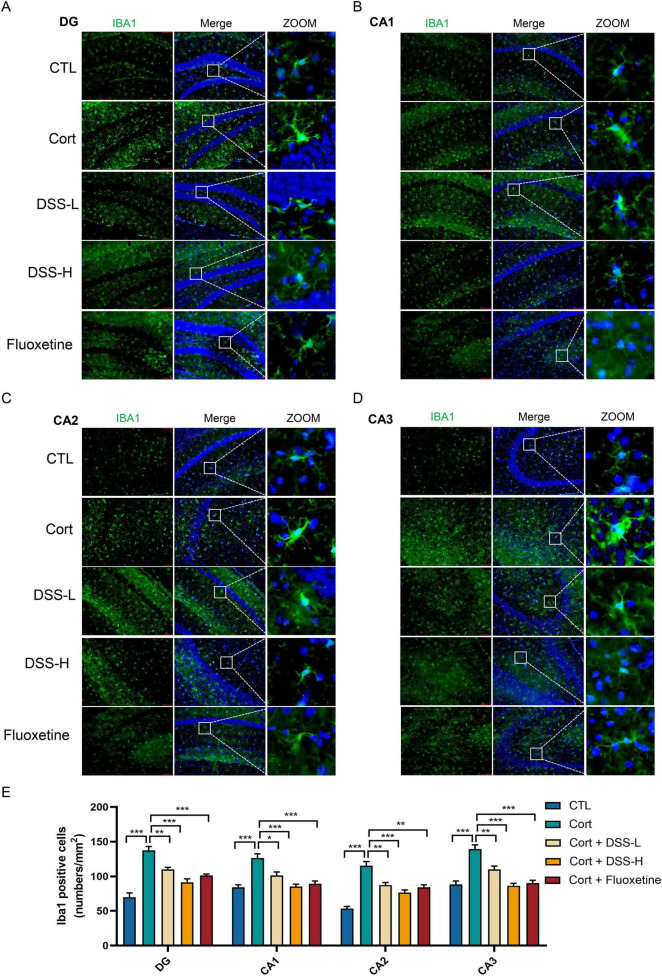
DSS suppresses hippocampal Neuroinflammation in mice. **(A)** Staining using coronal section of mouse, representative images labeled with IBA1 was shown in the dentate gyrus (DG) region of the mice’s hippocampus. **(B)** Representative images of IBA1 labeling in the CA1 region of the mouse hippocampus. **(C)** Representative images of IBA1 labeling in the CA2 region of the mouse hippocampus. **(D)** Representative images of IBA1 labeling in the CA3 region of the mouse hippocampus. **(E)** Quantification of microglia labeled with IBA1 in the mouse hippocampus. Data are expressed as Mean ± Sem, *n* = 3, *P < 0.05, **P < 0.01, ***P < 0.001.

Our findings indicate that DSS alleviates depressive-like behaviors in mice by regulating hippocampal neurogenesis and neuroinflammation. To further elucidate this mechanism, we conducted immunofluorescence staining of microglia in hippocampal subregions, including the dentate gyrus (DG), CA1, CA2, and CA3. Results showed a significant increase in microglial numbers in the model group, with the majority of cells displaying an amoeboid (activated) morphology. In contrast, DSS and fluoxetine treatment significantly decreased microglial numbers and restored these cells from an activated (amoeboid) to a resting state (Figures 5A–E). Collectively, these findings suggest that DSS, at least partially, exerts its antidepressant effects by suppressing microglial activation and reducing hippocampal neuroinflammation.

**FIGURE 6:**
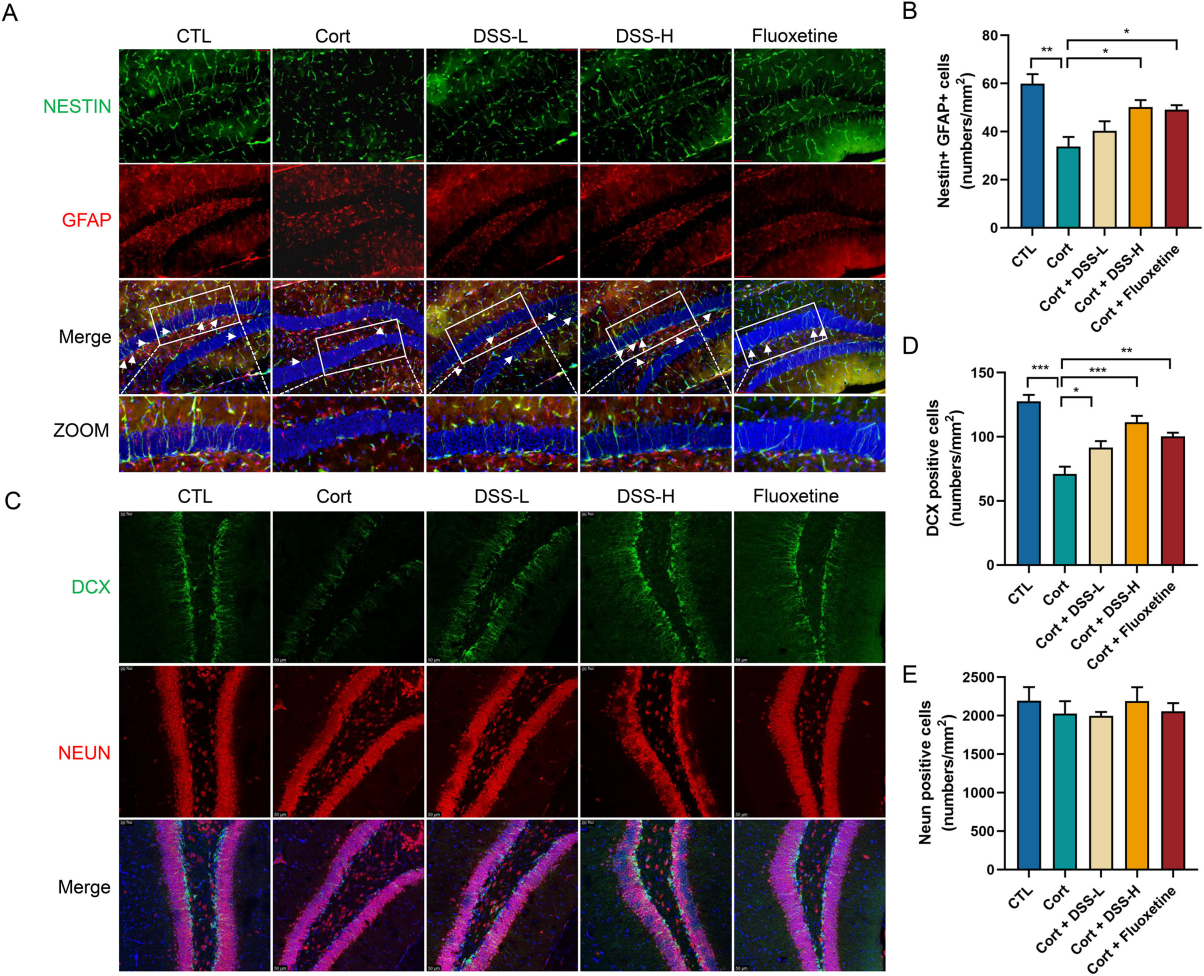
DSS enhances hippocampal neurogenesis in mice. **(A)** Staining using coronal section of mice, representative images labeled with NESTIN and GFAP were shown in the hippocampus of mice. **(B)** The number of NSCs co-labeled with NESTIN and GFAP was quantified in the mouse hippocampus. **(C)** Representative images of co-labeling with DCX and NEUN in the DG region of the hippocampus of mouse. **(D)** The number of DCX-labeled newborn neurons was quantified in the hippocampus of mouse. **(E)** The number of NEUN-labeled mature neurons was quantified in the hippocampus of mouse. Data are expressed as Mean ± Sem, *n* = 3, *P < 0.05, **P < 0.01, ***P < 0.001.

Additionally, the number of NSCs was significantly decreased in corticosterone-induced mice. However, this decrease was effectively reversed by high-dose DSS and fluoxetine treatment (Figures 6A, B). To evaluate neurogenesis, doublecortin (DCX) was employed as a marker for newly generated neurons. Corticosterone administration markedly reduced the number of DCX-positive cells in the hippocampus, whereas both DSS and fluoxetine significantly ameliorated this reduction (Figures 6C, D). The number of mature hippocampal neurons remained unchanged following treatment with corticosterone, DSS, or fluoxetine (Figures 6C–E). Collectively, these results indicate that DSS promotes hippocampal neurogenesis by enhancing the survival and differentiation of NSCs, thereby counteracting the negative effects of corticosterone.

### 3.6 DSS exerts its antidepressant effect by regulating neuroinflammation and promoting hippocampal neurogenesis

**FIGURE 7:**
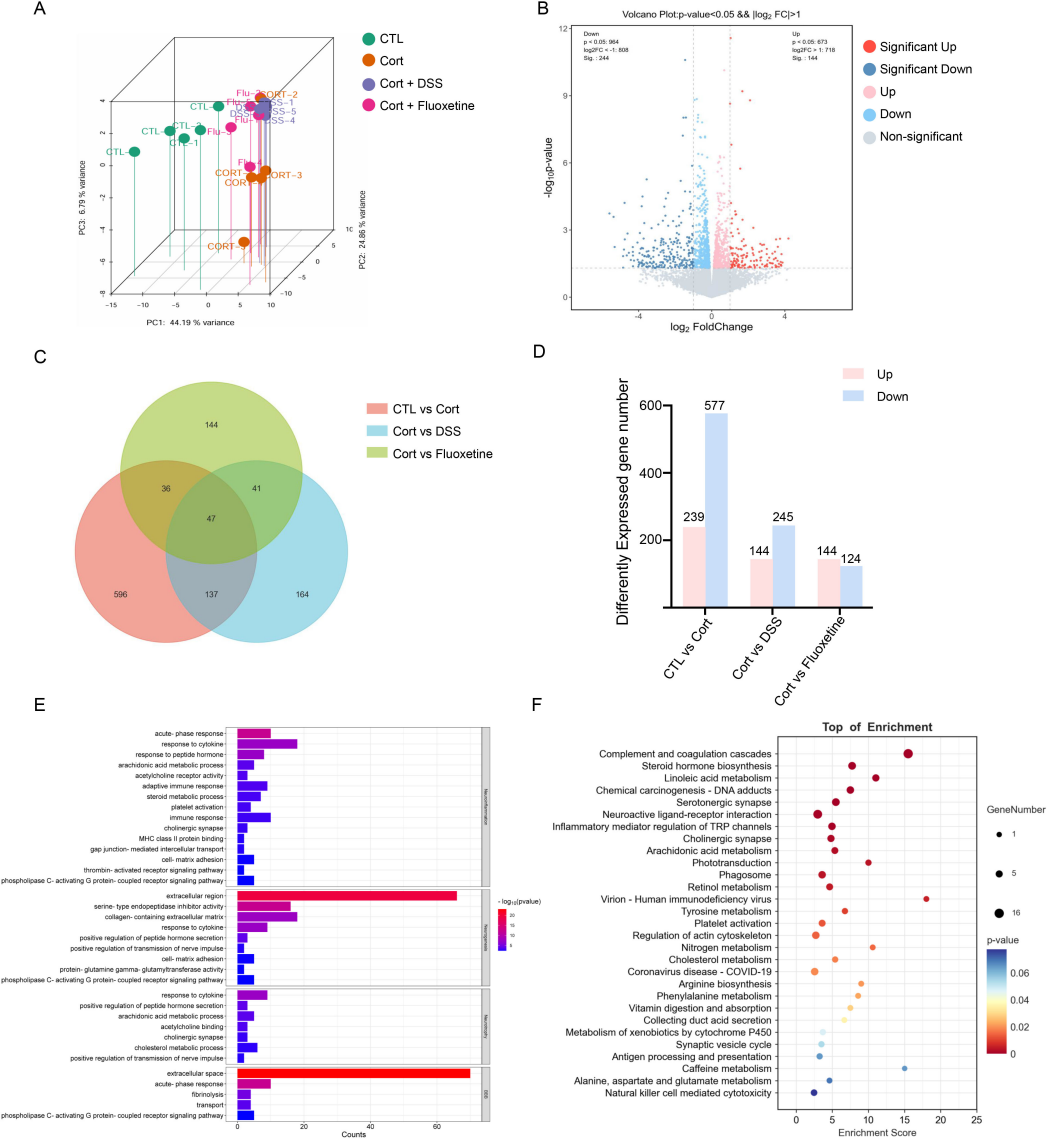
The results of differentially expressed genes (DEGs) analyzed by RNA-seq in the mouse hippocampus treated with corticosterone or DSS. **(A)** Principal component analysis (PCA) 3D plot of RNA sequencing data. Representative control group (CTL), corticosterone-treated group (Cort), corticosterone+DSS-treated group (Cort+DSS), Representative corticosterone+fluoxetine-treated group (Cort+Fluoxetine). **(B)** Volcano plots of changes in expression and significance of the same gene among different treatment groups. **(C)** Venn diagram demonstrating the overlapping situation of differentially expressed genes among different treatment groups. **(D)** The number of differentially expressed genes among different treatment groups. The pink bar represents the number of up-regulated genes and the blue bar represents the number of down-regulated genes. **(E)** Visualization of the GO term enrichment analysis. **(F)** Visualization of the KEGG pathway enrichment analysis.

To further validate our predictions and findings, we conducted RNA-seq on hippocampal tissues from mice treated with high-dose DSS. Compared with the depression model group, 144 genes were significantly upregulated and 245 genes were significantly downregulated in the DSS-treated group. By intersecting differentially expressed genes (DEGs) from the control and model groups, we identified 184 overlapping genes (Figures 7A–D).

Subsequent GO enrichment analysis of DEGs between the DSS-treated and corticosterone groups revealed significant enrichment of terms associated with neuroinflammation and/or neurogenesis. Key enriched terms included “acute-phase response,” “cytokine response,” “immune response,” and “serine-type endopeptidase inhibitor activity” (Figure 7E). And KEGG enrichment analysis revealed significant enrichment of terms such as “Neuroactive ligand - receptor interaction” and “Cholinergic synapse” (Figure 7F). These findings suggest that DSS may exert its antidepressant effects by modulating neuroinflammatory processes and promoting hippocampal neurogenesis.

### 3.7 DSS modulates the TLR4/NF-κB p65, JAK2/STAT3, and AKT-GSK3β signaling pathways, thereby affecting hippocampal neuroinflammation and the proliferation of NSCs

**FIGURE 8:**
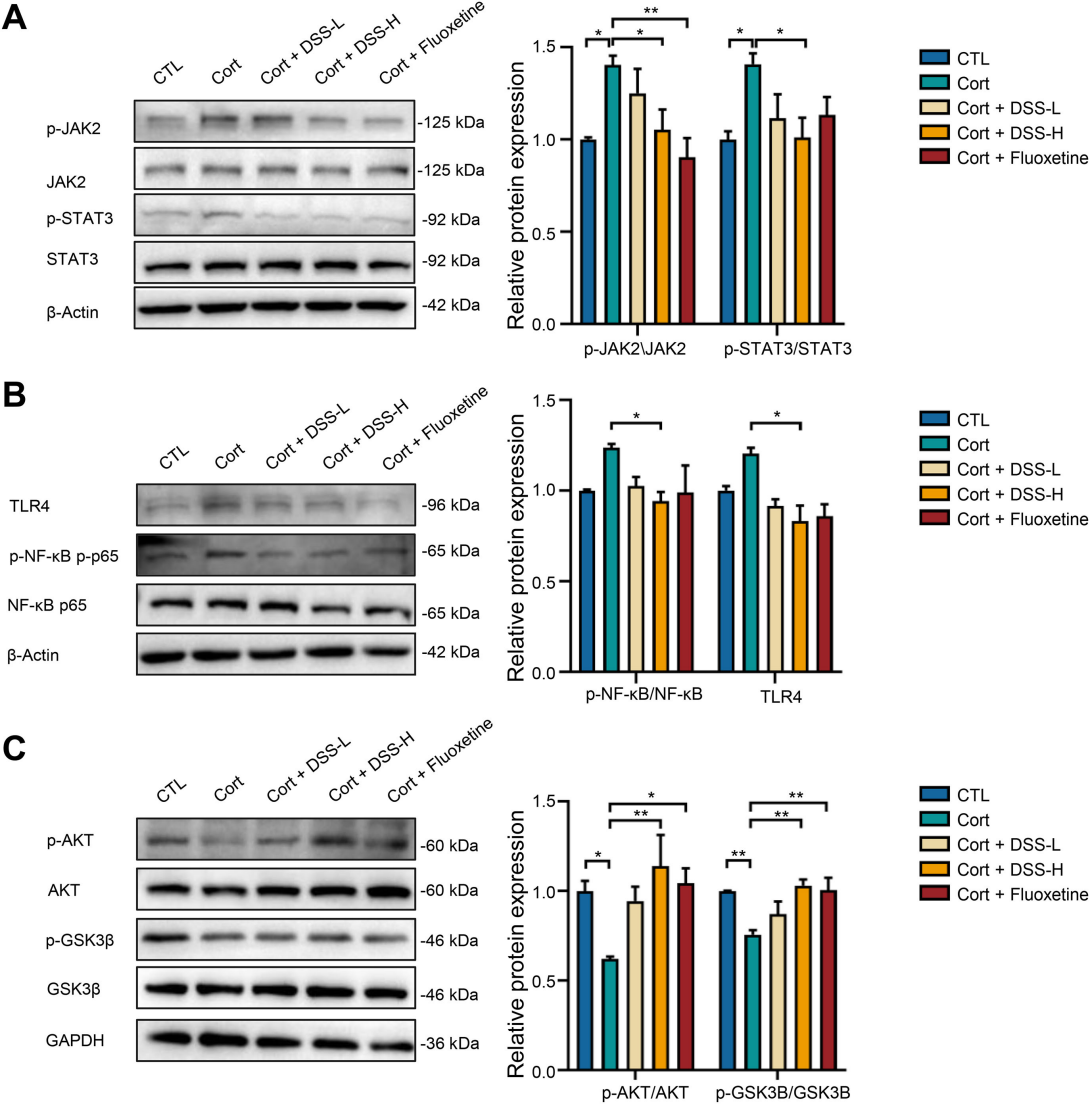
Western blotting was used to detect protein expression in mice hippocampal tissue (*n* = 3). **(A)** Western blotting was used to detect the protein levels of p-JAK2, JAK2, p-STAT3, STAT3 in mouse hippocampus. **(B)** Western blotting was used to detect the protein levels of TLR4, p-NF-κB p65, NF-κB p65 in mouse hippocampus. **(C)** Western blotting was used to detect the protein levels of p-AKT, AKT, p-GSK3β, GSK3β in mouse hippocampus. Data are expressed as Mean ± Sem, *n* ≥ 3, *P < 0.05, **P < 0.01.

Building on RNA-sequencing and molecular docking results, we further elucidated the mechanistic actions of DSS in depression by assessing hippocampal protein expression in mice. Western blot analysis (Figure 8A) revealed that, relative to the control (CTL) group, corticosterone-treated mice exhibited significantly elevated hippocampal TLR4 protein expression and a higher p-NF-κB p65/NF-κB p65 ratio. This upregulation suggests activation of the TLR4/NF-κB p65 signaling pathway. Notably, DSS treatment reversed this trend. Collectively, these data indicate that the TLR4/NF-κB p65 signaling pathway may mediate DSS’s inhibitory effects on hippocampal neuroinflammation in depressive mice.

The JAK/STAT signaling pathway, a critical mediator of cytokine transduction, is involved in inflammatory responses and has been recognized as a key player in central nervous system (CNS) disorders. In corticosterone-induced mice, elevated hippocampal phosphorylation levels of Janus kinase 2 (JAK2) and signal transducer and activator of transcription 3 (STAT3) were observed (Figure 8B). DSS treatment inhibited this phosphorylation. Thus, DSS may regulate inflammatory cytokine production and exert anti-inflammatory effects via activation of the JAK2/STAT3 pathway.

Additionally, the AKT-GSK3β signaling pathway is strongly implicated in depression. Previous studies have established its critical role in nervous system cell development, growth, and survival—including the repair of damaged hippocampal neural progenitor cells, which supports neuronal remodeling and neurotransmitter homeostasis. Western blot analysis (Figure 8C) revealed that corticosterone significantly decreased hippocampal p-AKT (phosphorylated AKT) levels and reduced GSK3β phosphorylation, consistent with increased AKT and GSK3β expression. DSS treatment reversed the p-GSK3β/GSK3β and p-AKT/AKT ratios. Collectively, these findings suggest that DSS alleviates CNS inflammation and depressive symptoms by modulating the TLR4/NF-κB p65, AKT-GSK3β, and JAK2/STAT3 signaling pathways.

## 4 Discussion

Depression, a severe mental disorder posing a major threat to human health, is characterized by complex pathogenesis and limited therapeutic options, remaining a critical challenge in psychiatric medicine. The current paucity of safe and effective therapeutic agents highlights the urgent need for innovative treatment strategies. TCM, a treasure trove of China’s ancient medical wisdom with millennia-long development and rich cultural heritage, provides distinctive multi-target therapeutic advantages in contemporary medical practice. Its distinctive polypharmacological properties offer novel insights into managing complex diseases. However, the inherent complexity of TCM formulas—defined by multiple components, targets, and pathways—poses substantial challenges to conventional experimental approaches in elucidating their synergistic mechanisms. This study adopts an integrative methodology integrating network pharmacology, RNA-seq technology, and experimental validation to explore depression-associated mechanisms, including neuroinflammation, and neurogenesis. Centering on corticosterone-induced depressive-like behaviors in mouse models, our goal is to elucidate the mechanistic basis of DSS in treating depression. Our findings are anticipated to open new avenues for the clinical management of depression via the innovative application of TCM-derived therapeutic strategies.

Dysregulation of amino acid and lipid metabolism has been demonstrated to impair hippocampal neurogenesis, neuroinflammation, and mood regulation (28). Our RNA sequencing analysis identified significant alterations in gene expression associated with amino acid metabolism, lipid metabolism, neuroinflammation, and neurogenesis in corticosterone-induced depressive mouse models. These findings not only confirm the critical associations between these biological processes and depression pathogenesis but also highlight complex interrelationships among them. Further investigations are required to fully elucidate their roles in the depression development and progression.

Previous studies have established that amino acid metabolic imbalances and lipid dysregulation represent critical biological processes in depression pathogenesis (29, 30). As precursors for neurotransmitter synthesis, amino acid metabolic alterations directly modulate neuronal signaling by altering neurotransmitter production (31, 32). Our RNA-seq analysis revealed that corticosterone regulates amino acid metabolism, notably altering serine, arginine, and tyrosine metabolic pathways. Similarly, lipid metabolic abnormalities impair receptor function and intracellular signaling pathways, disrupting normal neural physiology and contributing to depressive pathology (33, 34). GO analysis identified significant enrichment of biological processes in corticosterone-induced depressive mouse models, including inflammatory responses, oxidative stress, glucose/lipid metabolism, and Blood-Brain Barrier (BBB) regulation in hippocampal tissues. Notably, we observed significant alterations in Natural Killer T (NKT) cell differentiation. While NKT cells are rarely present in the brain under physiological conditions, they can infiltrate brain regions through the compromised BBB during inflammatory conditions. The BBB, a specialized biological barrier, primarily protects the CNS from blood-derived toxins while limiting immune cell infiltration and maintaining CNS homeostasis (35). However, neuroinflammation enhances BBB permeability, facilitating NKT cell migration to inflammatory brain regions (36). This phenomenon indirectly suggests BBB dysfunction and hippocampal neuroinflammation progression in depression models.

Our study further demonstrated impaired microglial function in corticosterone-induced depressive mouse models, characterized by hippocampal inflammatory responses, disrupted neurogenesis, and significantly reduced counts of newborn cells. RNA-seq analysis revealed dysregulation of CD36 expression—a key gene linked to microglial phagocytosis. As a core function of microglia, phagocytic activity plays a critical role in maintaining cerebral homeostasis through the clearance of metabolic waste, cellular debris, misfolded proteins, and pathogens (37). This functional impairment is likely to contribute to pathological neuroinflammation and suppressed neurogenesis, both of which are recognized as pivotal drivers of depression progression. However, whether microglial phagocytic capacity is fundamentally impaired in this specific context warrants further experimental validation.

DSS, a classical herbal formula with deep roots in traditional Chinese medical texts, has a long-standing history of medicinal use. Composed of six herbs—*Angelica sinensis* (Danggui), *Paeonia lactiflora* (Shaoyao), *Ligusticum chuanxiong* (Chuanxiong), *Poria cocos* (*Fuling*), *Atractylodes macrocephala* (*Baizhu*), and *Alisma orientale* (*Zexie*), this formulation holds enduring significance in TCM. These botanicals are not only widely used in clinical TCM practice but also integrated into daily life, with many serving dual roles as both culinary ingredients and medicinal components. For dietary use, DSS is traditionally prepared by decocting its six constituent herbs, with the resulting decoction combined with rice to make DSS congee (herbal rice porridge). As documented in *Traditional Chinese Medicine Otolaryngology*, the DSS decoction primarily consists of these six herbs, namely *Angelica sinensis*, *Paeonia lactiflora*, *Poria cocos*, *Atractylodes macrocephala*, *Alisma orientalis*, and *Ligusticum chuanxiong*, and is appropriately combined with other herbal medicines. It remains highly prevalent in daily diets. Beyond formal formulations, individual DSS components are also incorporated into daily cuisine: *Poria cocos* (Fuling) is frequently used in pastries such as Fuling cakes or added to congee, while *Atractylodes macrocephala* (Baizhu) not only enhances the flavor profile of dishes but also provides health benefits. The formula’s strong safety profile, commercial availability, and accessibility further support its integration into modern dietary practices. The formula’s strong safety profile, wide commercial availability, and ease of access further support its integration into modern dietary practices. This unique combination of historical validation, culinary versatility, and practical accessibility positions DSS as a valuable resource for preventive healthcare and wellness maintenance via daily nutritional intake. However, this study utilized TCMSP, DrugBank, and related databases for compound screening, target identification, and network construction. These databases may not fully cover all compounds and might inadequately reflect the pharmacokinetic characteristics or synergistic effects of traditional Chinese medicine (TCM) formulations, thus presenting certain limitations.

Our experimental findings indicate that DSS alleviates central neuroinflammation and regulates NSC proliferation, potentially via coordinated modulation of the TLR4/NF-κB p65, JAK2-STAT3, and AKT-GSK3β signaling axes. In corticosterone-induced mice, persistent activation of the TLR4/NF-κB p65, JAK2-STAT3, and AKT-GSK3β pathways was detected, aligning with neuroinflammatory cascades associated with depression. Notably, intragastric DSS administration reversed these abnormalities, as demonstrated by the restoration of corticosterone-disrupted p-GSK3β/GSK3β and p-AKT/AKT phosphorylation ratios, inhibition of JAK2-STAT3 hyperphosphorylation, and concurrent amelioration of depressive-like behavioral phenotypes. Collectively, these findings suggest that DSS-mediated multi-pathway modulation represents a plausible mechanism underlying its therapeutic efficacy in corticosterone-induced depression models.

Overall, this study identified alterations in genes associated with neuroinflammation, and neural regeneration during depression, and demonstrated that DSS exerts antidepressant effects through the regulation of neuroinflammation-and neurotrophic-related gene expression, providing novel theoretical foundations and potential targets for developing depression treatment strategies. Nevertheless, this study has certain limitations. First, the research was conducted exclusively in a mouse model. The pharmacokinetic and pharmacodynamic profiles of DSS in humans, as well as its efficacy and safety in treating human depression, require validation through clinical trials. Second, the complex interactive regulatory mechanisms among depression-related pathways, as well as the precise regulatory effects of DSS on upstream and downstream molecules within these pathways, remain poorly understood. We will further investigate the mechanistic actions of DSS’s bioactive components to comprehensively elucidate the complex interrelationships among DSS, its bioactive constituents, and the pathophysiological mechanisms underlying depression.

## 5 Conclusion

Collectively, DSS exhibits potent antidepressant effects in corticosterone-induced depressive mouse models by regulating hippocampal neurogenesis and attenuating neuroinflammation. Through its multi-component composition comprising β-sitosterol, 3β-acetoxyatractylone, and stigmasterol, DSS potentially targets the TLR4/NF-κB p65, JAK2/STAT3, and AKT-GSK3β signaling pathways. DSS restores NSC counts and attenuates microglial activation in the mouse hippocampus. These findings not only validate the traditional application of DSS in mood disorders but also provide a scientific rationale for its development as a potential complementary therapeutic agent for depression.

## Data Availability

The original contributions presented in the study are publicly available. This data can be found here: https://www.ncbi.nlm.nih.gov/bioproject/?term=PRJNA1337884.

## Footnotes

1
https://old.tcmsp-e.com/tcmsp.php

2
https://go.drugbank.com

3
http://swisstargetprediction.ch

4
https://db.idrblab.net

5
https://bioinfogp.cnb.csic.es/tools/venny/index.html

6
https://string-db.org

7
https://david.ncifcrf.gov

8
https://www.rcsb.org
