# Correction: Danggui Shaoyao San attenuates depressive-like behaviors in mice via TLR4/NF-κB p65/JAK-STAT3/AKT-GSK3β signaling pathways: modulation of hippocampal neurogenesis and neuroinflammation

**DOI:** 10.3389/fnut.2025.1761855

**Published:** 2026-01-05

**Authors:** Chuan-Feng Tang, Fan Li, Lu-Han Ma, Qiao-Na Wang, Peng-Fei Xie, Lang Xiang, Yu-Jie Zhu, Yue-Yao Wang, Yi-Zhu Zhang, Jun-Jie Shi, Sheng-Jie Li, Jian-Mei Li

**Affiliations:** 1 State Key Laboratory of Technologies for Chinese Medicine Pharmaceutical Process Control and Intelligent Manufacture, Department of Pharmacy, Nanjing University of Chinese Medicine, Nanjing, China; 2 School of Food Science, Nanjing Xiaozhuang University, Nanjing, China; 3 School of Food Science and Pharmaceutical Engineering, Nanjing Normal University, Nanjing, China

**Keywords:** DSS, depression, hippocampal neurogenesis, hippocampal inflammation, TLR4/NF-κB p65, JAK2/STAT3 and AKT-GSK3β signaling pathways

There was a mistake in Figure 5 as published. The images for CTL and Flouxetine groups in the CA2 region were incorrect during the preparation. The corrected Figure 5 and its caption appear below.

**Figure 5:**
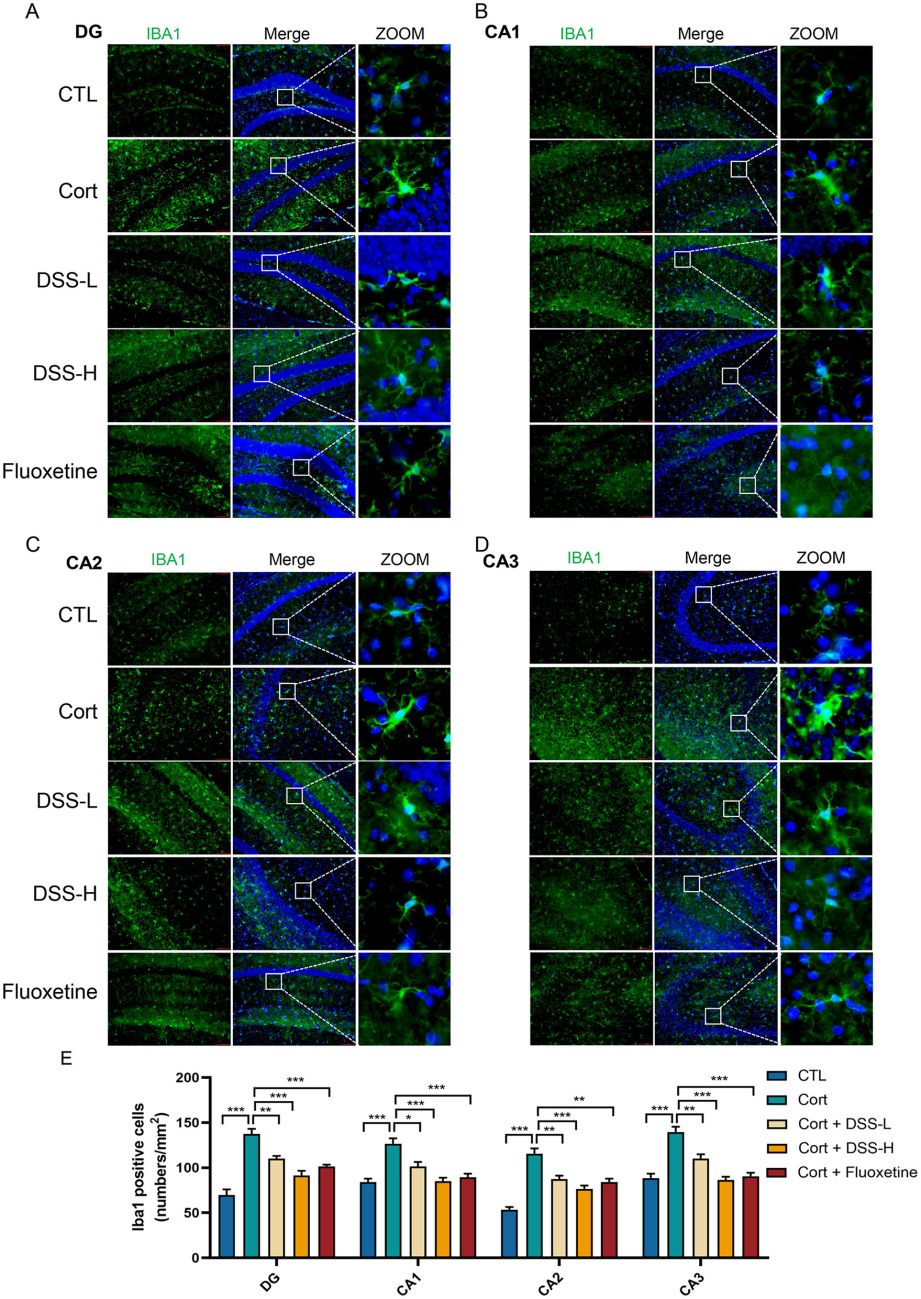
DSS suppresses hippocampal neuroinflammation in mice. **(A)** Staining using coronal section of mouse, representative images labeled with IBA1 was shown in the dentate gyrus (DG) region of the mice's hippocampus. **(B)** Representative images of IBA1 labeling in the CA1 region of the mouse hippocampus. **(C)** Representative images of IBA1 labeling in the CA2 region of the mouse hippocampus. **(D)** Representative images of IBA1 labeling in the CA3 region of the mouse hippocampus. **(E)** Quantification of microglia labeled with IBA1 in the mouse hippocampus. Data are expressed as Mean ± SEM, *n* = 3, ^*^P < 0.05, ^**^P < 0.01, ^***^P < 0.001.

An incorrect number was provided for the grant Natural Science Foundation of Jiangsu Province. The incorrect number was written as, “012071002966, C-FT”. The correct number is “BK20250770, C-FT”.

The original version of this article has been updated.

