# Supplementary material for: Danggui Shaoyao San attenuates depressive-like behaviors in mice via TLR4/NF-κB p65/JAK-STAT3/AKT-GSK3β signaling pathways: modulation of hippocampal neurogenesis and neuroinflammation
Source: Front Nutr. 2025 Oct 20;12:1652968. doi: 10.3389/fnut.2025.1652968 (PMC12580595; doi:10.3389/fnut.2025.1652968)
Supplement: Supplementary file 1 [file Supplementary_file_1.docx]

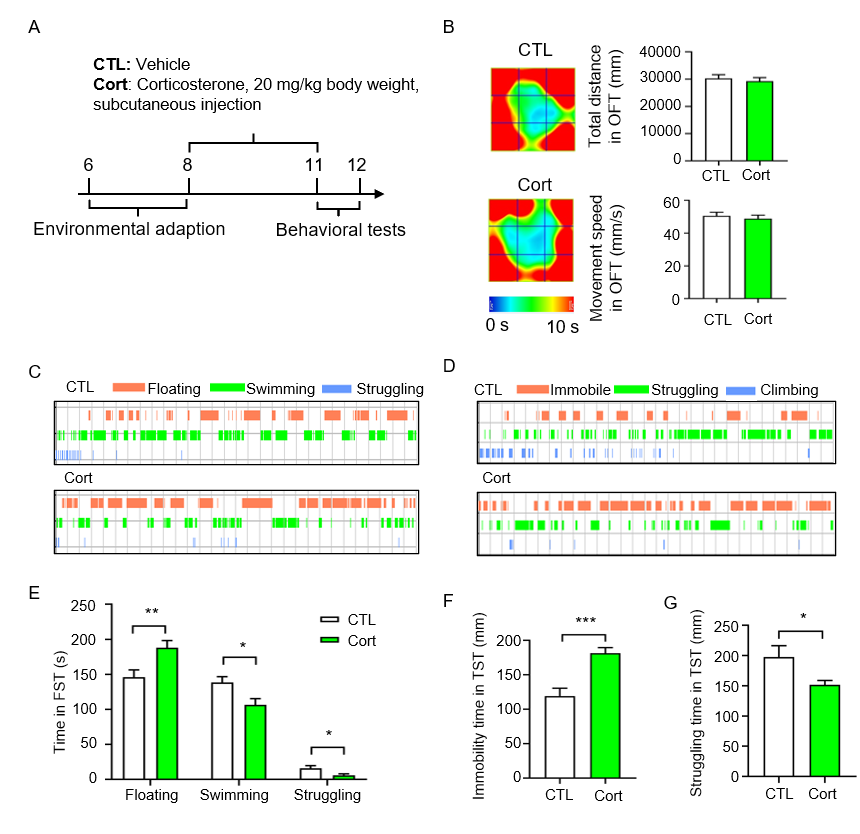


**Figure S1** Corticosterone can induce depressive-like behaviors in mice. (**A**) Experimental timeline of corticosterone intervention and behavioral tests. (B) Representative heatmaps (left) and quantitative data (right) depicting total distance traveled and movement speed in the open field test (OFT) for CTL and Cort groups. (C) Behavioral pattern diagrams from the forced swim test (FST) for CTL and Cort groups, illustrating floating, swimming, and struggling behaviors over time. (D) Behavioral pattern diagrams from the tail suspension test (TST) for CTL and Cort groups, showing immobile, struggling, and climbing behaviors over time. (E) Statistical results of the FST. (F) Statistical results of the immobile time in TST. (**G**) Statistical results of the struggling time in TST. Data are expressed as Mean ± Sem, n≥6, **P* < 0.05, ***P* < 0.01, ****P* < 0.001.


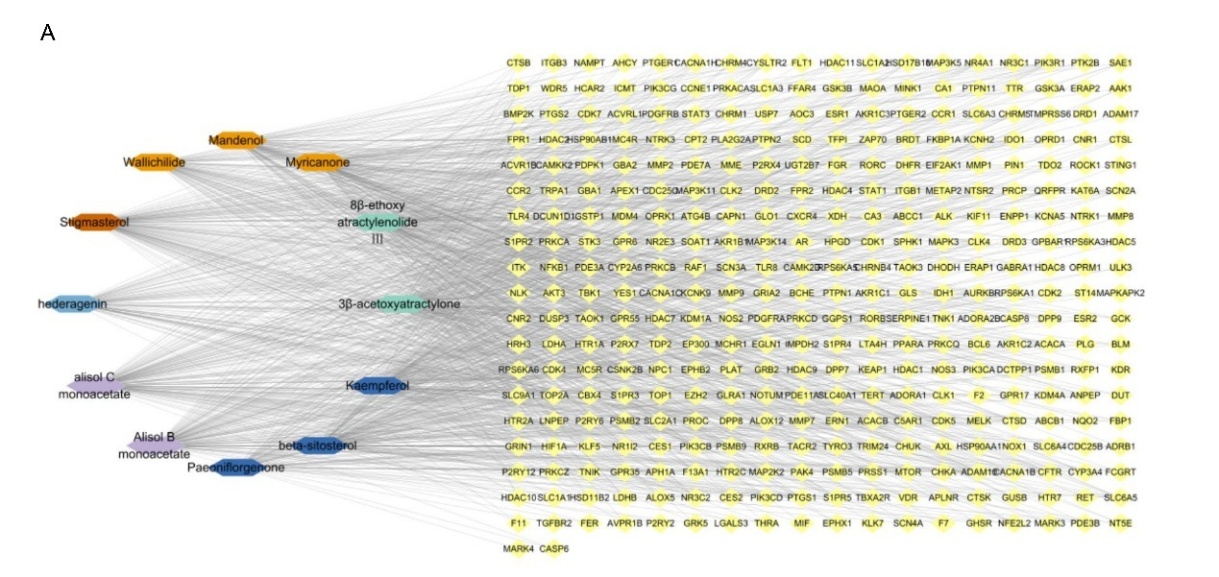


**Figure S2** The herbs-compound-target network Showing 344 Overlapping Targets Between 12 Bioactive Components of DSS and depression.


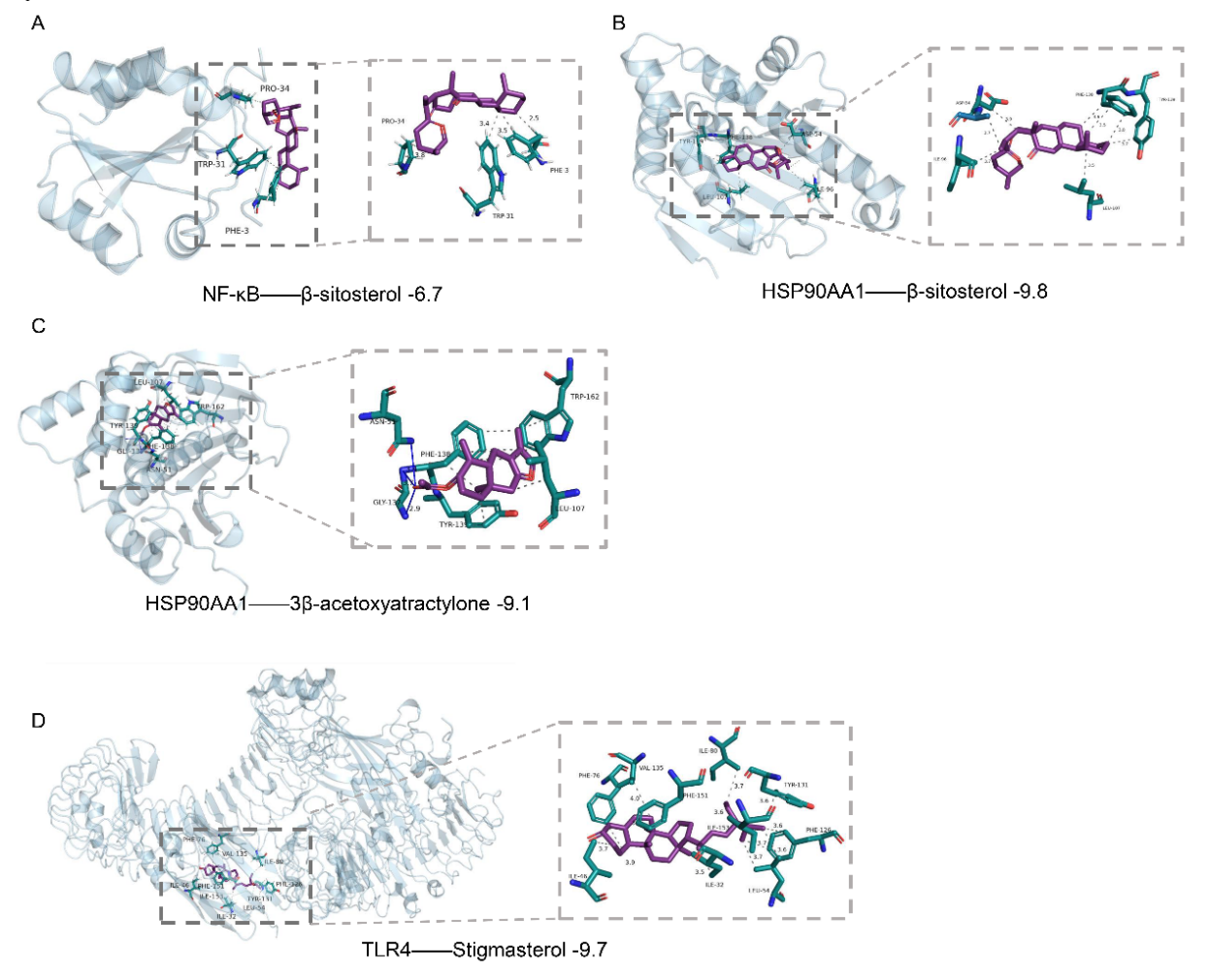


**Figure S3** Represents of molecular docking visualization of key compounds and key targets (A) NF-κB and β-sitosterol (binding energy = -6.7 kcal/mol); (B) HSP90AA1 and β-sitosterol (binding energy = -9.6 kcal/mol); (C) HSP90AA1 and 3β-acetoxyatractylone (binding energy = -9.1 kcal/mol); (D) TLR4 and stigmasterol (binding energy = -9.7 kcal/mol)

**Table S1.** The ID, OB and DL of compounds in Danggui Shaoyao San (DSS)**.**

| **Mol ID** | **Molecule Name** | **MV** | **AIogP** | **OB(%)** | **DL** | **TCM** |
| --- | --- | --- | --- | --- | --- | --- |
| MOL001494 | Mandenol | 308.56 | 6.99 | 42 | 0.19 | *Ligusticum chuanxiong Hot.* (Chuanxiong) |
| MOL002151 | senkyunone | 326.52 | 6.32 | 47.66 | 0.24 |  |
| MOL002140 | Perlolyrine | 264.3 | 3.2 | 65.95 | 0.27 |  |
| MOL002135 | Myricanone | 356.45 | 4.1 | 40.6 | 0.51 |  |
| MOL000433 | FA | 441.45 | 0.01 | 68.96 | 0.71 |  |
| MOL002157 | wallichilide | 412.57 | 4.82 | 42.31 | 0.71 |  |
| MOL000359 | sitosterol | 414.79 | 8.08 | 36.91 | 0.75 |  |
| MOL000358 | beta-sitosterol | 414.79 | 8.08 | 36.91 | 0.75 | *Angelica Sinensis (Oliv.) Diels* (Danggui) |
| MOL000449 | Stigmasterol | 412.77 | 7.64 | 43.83 | 0.76 |  |
| MOL001910 | 11alpha,12alpha-epoxy-3beta-23-dihydroxy-30-norolean-20- en-28,12beta-olide | 470.71 | 3.91 | 64.77 | 0.38 | *Paeonia lactiflora* (Shaoyao) |
| MOL001918 | paeoniflorgenone | 318.35 | 0.79 | 87.59 | 0.37 |  |
| MOL001919 | (3S,5R,8R,9R,10S,14S)-3,17-dihydroxy-4,4,8,10,14-pentamethyl-2,3,5,6,7,9-hexahydro-1H-cyclopenta[a]phenanthrene-15,16-dione | 358.52 | 2.69 | 43.56 | 0.53 |  |
| MOL001921 | Lactiflorin | 462.49 | -0.57 | 49.12 | 0.8 |  |
| MOL001924 | paeoniflorin | 480.51 | -1.28 | 53.87 | 0.79 |  |
| MOL001925 | paeonifloin_qt | 318.35 | 0.46 | 68.18 | 0.4 |  |
| MOL001928 | albiflorin_qt | 318.35 | 0.42 | 66.64 | 0.33 |  |
| MOL001930 | benzoyl paeoniflorin | 584.62 | 0.81 | 31.27 | 0.75 |  |
| MOL000211 | Mairin | 456.78 | 6.52 | 55.38 | 0.78 |  |
| MOL000358 | beta-sitosterol | 414.79 | 8.08 | 36.91 | 0.75 |  |
| MOL000359 | sitosterol | 414.79 | 8.08 | 36.91 | 0.75 |  |
| MOL000422 | kaempferol | 286.25 | 1.77 | 41.88 | 0.24 |  |
| MOL000492 | (+)-catechin | 290.29 | 1.92 | 54.83 | 0.24 |  |
| MOL000020 | 12-senecioyl-2E,8E,10E-atractylentriol | 312.39 | 2.5 | 62.4 | 0.22 | *Ptratylodes Macrocephala Koidz.* (Baizhu) |
| MOL000021 | 14-acetyl-12-senecioyl-2E,8E,10E-atractylentriol | 355.44 | 3.21 | 60.31 | 0.31 |  |
| MOL000022 | 14-acetyl-12-senecioyl-2E,8Z,10E-atractylentriol | 356.45 | 3.54 | 63.37 | 0.3 |  |
| MOL000028 | α-Amyrin | 426.8 | 7.35 | 39.51 | 0.76 |  |
| MOL000033 | (3S,8S,9S,10R,13R,14S,17R)-10,13-dimethyl-17-[(2R,5S)-5-propan-2-yloctan-2-yl]-2,3,4,7,8,9,11,12,14,15,16,17-dodecahydro-1H-cyclopenta[a]phenanthren-3-0l | 428.82 | 8.54 | 36.23 | 0.78 |  |
| MOL000049 | 3β-acetoxyatractylone | 274.39 | 3.39 | 54.07 | 0.22 |  |
| MOL000072 | 8β-ethoxy atractylenolide Ⅲ | 276.41 | 3.68 | 35.95 | 0.21 |  |
| MOL000359 | sitosterol | 414.79 | 8.08 | 36.91 | 0.75 | Alisma orientale (sam.) juzep (Zexie) |
| MOL000830 | Alisol B | 472.78 | 4.64 | 34.47 | 0.82 |  |
| MOL000831 | Alisol B monoacetate | 514.82 | 5.02 | 35.58 | 0.81 |  |
| MOL000832 | alisol,b,23-acetate | 446.74 | 3.82 | 32.52 | 0.82 |  |
| MOL000849 | 16β-methoxyalisol B monoacetate | 544.85 | 4.33 | 32.43 | 0.77 |  |
| MOL000853 | alisol B | 444.72 | 3.76 | 36.76 | 0.82 |  |
| MOL000854 | alisol C | 486.76 | 3.5 | 32.7 | 0.82 |  |
| MOL000856 | alisol C monoacetate | 514.77 | 3.67 | 33.06 | 0.83 |  |
| MOL002464 | 1-Monolinolein | 354.59 | 5.59 | 37.18 | 0.3 |  |
| MOL000862 | [(1S,3R)-1-[(2R)-3,3-dimethyloxiran-2-yl]-3-[(5R,8S,9S,10S,11S,14R)-11-hydroxy-4,4,8,10,14-pentamethyl-3-0Xo-1,2,5,6,7,9,11,12,15,16-decahydrocyclopenta[a]phenanthren-17-yl]butyl]acetate | 514.82 | 514.82 | 35.58 | 0.81 |  |
| MOL000273 | (2R)-2-[(3S,5R,10S,13R,14R,16R,17R)-3,16-dihydroxy-4,4,10,13,14-pentamethyl-2,3,5,6,12,15,16,17-octahydro-1H-cyclopenta[a]phenanthren-17-y]-6-methylhept-5-enoic acid | 470.76 | 5.41 | 30.93 | 0.81 | *Poria cocos (Schw.)* *Wolf* (Fuling) |
| MOL000275 | trametenolic acid | 456.78 | 7.03 | 38.71 | 0.8 |  |
| MOL000276 | 7,9(11)-dehydropachymic acid | 526.83 | 6.1 | 35.11 | 0.81 |  |
| MOL000279 | Cerevisterol | 430.74 | 5.15 | 37.96 | 0.77 |  |
| MOL000280 | (2R)-2-[(3S,5R,10S,13R,14R,16R,17R)-3,16-dihydroxy-4,4,10,13,14-pentamethyl-2,3,5,6,12,15,16,17-octahydro-1H-cyclopenta[a]phenanthren-17-yl]-5-isopropyl-hex-5-enoic acid | 484.79 | 5.72 | 31.07 | 0.82 |  |
| MOL000282 | ergosta-7,22E-dien-3beta-ol | 398.74 | 7.18 | 43.51 | 0.72 |  |
| MOL000283 | Ergosterol peroxide | 430.74 | 7.17 | 40.36 | 0.81 |  |
| MOL000285 | (2R)-2-[(5R,10S,13R,14R,16R,17R)-16-hydroxy-3-keto-4,4,10,13,14-pentamethyl-1,2,5,6,12,15,16,17-octahydrocyclopenta[a]phenanthren-17-yl]-5-isopropyl-hex-5-enoicacid | 482.77 | 5.68 | 38.26 | 0.82 |  |
| MOL000287 | 3beta-Hydroxy-24-methylene-8-lanostene-21-oic acid | 470.81 | 7.33 | 38.7 | 0.81 |  |
| MOL000289 | pachymicacid | 528.85 | 6.54 | 33.63 | 0.81 |  |
| MOL000290 | Poricoic acid A | 498.77 | 5.94 | 30.61 | 0.76 |  |
| MOL000291 | Poricoic acid B | 484.74 | 5.64 | 30.52 | 0.75 |  |
| MOL000292 | poricoicacid C | 482.77 | 7.11 | 38.15 | 0.75 |  |
| MOL000296 | hederagenin | 414.79 | 8.08 | 36.91 | 0.75 |  |
| MOL000300 | dehydroeburicoic acid | 453.75 | 6.35 | 44.17 | 0.83 |  |

**Table S2.** The key components of Danggui Shaoyao San (DSS)**.**

| **MOI ID** | **Key Components** | **TCM** | **PubChem CID** | **MV** | **AIogP** | **OB（%）** | **DL** |
| --- | --- | --- | --- | --- | --- | --- | --- |
| MOL001494 | Mandenol | Chuanxiong | 5282184 | 308.56 | 6.99 | 42 | 0.19 |
| MOL002135 | Myricanone |  | 161748 | 356.45 | 4.1 | 40.6 | 0.51 |
| MOL002157 | Wallichilide |  | 10873344 | 412.57 | 4.82 | 42.31 | 0.71 |
| MOL000449 | Stigmasterol | Danggui | 5280794 | 412.77 | 7.64 | 43.83 | 0.76 |
| MOL001918 | paeoniflorigenone | Shaoyao | 133475 | 318.35 | 0.79 | 87.59 | 0.37 |
| MOL000358 | β-sitosterol |  | 222284 | 414.79 | 8.08 | 36.91 | 0.75 |
| MOL000422 | Kaempferol |  | 5280863 | 286.25 | 1.77 | 41.88 | 0.24 |
| MOL000049 | 3β-acetoxyatractylone | Baizhu | 163082913 | 274.39 | 3.39 | 54.07 | 0.22 |
| MOL000072 | 8β-ethoxy atractylenolide Ⅲ |  | 5317226 | 276.41 | 3.68 | 35.95 | 0.21 |
| MOL000831 | Alisol B monoacetate | Zexie | 138756747 | 514.82 | 5.02 | 35.58 | 0.81 |
| MOL000856 | Alisol C monoacetate |  | 14036813 | 514.77 | 3.67 | 33.06 | 0.83 |
| MOL000296 | hederagenin | Fuling | 73299 | 414.79 | 8.08 | 36.91 | 0.75 |

**Table S3.** Main targets of 3β-acetoxyatractylone

| No. | target | No. | target | No. | target | No. | target |
| --- | --- | --- | --- | --- | --- | --- | --- |
| 1 | APEX1 | 27 | CACNA1B | 56 | HSD17B10 | 84 | AURKB |
| 2 | CLK4 | 28 | PIK3CA | 57 | NOS3 | 85 | HDAC11 |
| 3 | TOP2A | 29 | HDAC5 | 58 | PDGFRA | 86 | CCR1 |
| 4 | NFKB1 | 30 | PDE3A | 59 | TNK1 | 87 | HDAC9 |
| 5 | BLM | 31 | FBP1 | 60 | SLC9A1 | 88 | TDP1 |
| 6 | ACACA | 32 | SCN2A | 61 | PIK3CB | 89 | GRB2 |
| 7 | KDM1A | 33 | DRD1 | 62 | GRIN1 | 90 | DRD3 |
| 8 | CTSD | 34 | KLF5 | 63 | RORB | 91 | SCD |
| 9 | PRKCD | 35 | S1PR5 | 64 | FLT1 | 92 | PSMB9 |
| 10 | GPR55 | 36 | RXFP1 | 65 | CDC25C | 93 | WDR5 |
| 11 | CYP3A4 | 37 | PRCP | 66 | ACACB | 94 | CHRM4 |
| 12 | DUSP3 | 38 | SCN3A | 67 | PTPN2 | 95 | CDK2 |
| 13 | NR3C2 | 39 | CDK5 | 68 | HDAC4 | 96 | MAP3K5 |
| 14 | FPR1 | 40 | METAP2 | 69 | ITK | 97 | HDAC10 |
| 15 | TRIM24 | 41 | PSMB1 | 70 | MC4R | 98 | NTSR2 |
| 16 | NTRK3 | 42 | CACNA1H | 71 | ZAP70 | 99 | TACR2 |
| 17 | CLK2 | 43 | HDAC2 | 72 | HDAC7 | 100 | KEAP1 |
| 18 | CNR2 | 44 | NR1I2 | 73 | AHCY | 101 | AKR1C3 |
| 19 | ITGB1 | 45 | AR | 74 | PLA2G2A | 102 | SERPINE1 |
| 20 | ADAM10 | 46 | TFPI | 75 | NOS2 | 103 | EPHB2 |
| 21 | CSNK2B | 47 | CDK4 | 76 | CFTR | 104 | TBXA2R |
| 22 | C5AR1 | 48 | HIF1A | 77 | RAF1 | 105 | PIK3CD |
| 23 | CDK1 | 49 | FCGRT | 78 | PRKCB | 106 | LDHB |
| 24 | FPR2 | 50 | PTGS1 | 79 | NR4A1 | 107 | VDR |
| 25 | TLR4 | 51 | QRFPR | 80 | HSD11B2 | 108 | PTK2B |
| 26 | PIK3R1 | 52 | SAE1 | 81 | TLR8 | 109 | HSP90AB1 |
| 27 | APEX1 | 53 | GPBAR1 | 82 | DRD2 | 110 | CHRM5 |
| 28 | CLK4 | 54 | CYSLTR2 | 83 | RXRB |  |  |

**Table S4.** Main targets of 8β-ethoxy atractylenolide Ⅲ

| No. | target | No. | target | No. | target | No. | target |
| --- | --- | --- | --- | --- | --- | --- | --- |
| 1 | TRIM24 | 33 | SCD | 65 | HDAC11 | 97 | PRSS1 |
| 2 | HSD17B10 | 34 | ADAM10 | 66 | SLC9A1 | 98 | ANPEP |
| 3 | CTSD | 35 | IDO1 | 67 | PDE11A | 99 | HSD11B2 |
| 4 | OPRM1 | 36 | PTGS1 | 68 | ABCC1 | 100 | STK3 |
| 5 | KDM1A | 37 | CDK1 | 69 | CYP3A4 | 101 | HRH3 |
| 6 | CLK4 | 38 | MAP2K2 | 70 | TDP2 | 102 | DUT |
| 7 | CSNK2B | 39 | TLR4 | 71 | ACACA | 103 | GUSB |
| 8 | KLF5 | 40 | AR | 72 | CDC25C | 104 | PROC |
| 9 | APEX1 | 41 | PIK3CD | 73 | PIK3CB | 105 | HSP90AA1 |
| 10 | NFKB1 | 42 | PRKCB | 74 | P2RX7 | 106 | GRIN1 |
| 11 | DUSP3 | 43 | AHCY | 75 | PSMB9 | 107 | SLC40A1 |
| 12 | CNR2 | 44 | ROCK1 | 76 | ZAP70 | 108 | GCK |
| 13 | GPR55 | 45 | PRKCA | 77 | LDHB | 109 | BRDT |
| 14 | C5AR1 | 46 | HDAC4 | 78 | PTPN2 | 110 | CYSLTR2 |
| 15 | NR1I2 | 47 | F7 | 79 | GABRA1 | 111 | SLC6A4 |
| 16 | NTRK3 | 48 | PDGFRA | 80 | SCN3A | 112 | TNK1 |
| 17 | OPRD1 | 49 | VDR | 81 | MC4R | 113 | HCAR2 |
| 18 | CACNA1B | 50 | MMP9 | 82 | NOTUM | 114 | OPRK1 |
| 19 | KEAP1 | 51 | FPR2 | 83 | SCN2A | 115 | HDAC8 |
| 20 | HDAC10 | 52 | STAT3 | 84 | DHODH | 116 | PIK3CG |
| 21 | CDK5 | 53 | FCGRT | 85 | DPP9 | 117 | TERT |
| 22 | NR3C2 | 54 | ICMT | 86 | NT5E | 118 | CCR1 |
| 23 | RORB | 55 | METAP2 | 87 | KCNA5 | 119 | USP7 |
| 24 | TOP2A | 56 | PIN1 | 88 | APLNR | 120 | TACR2 |
| 25 | FPR1 | 57 | ITK | 89 | PRKCQ | 121 | SLC6A5 |
| 26 | AURKB | 58 | ACACB | 90 | MARK3 | 122 | MMP1 |
| 27 | CFTR | 59 | NR4A1 | 91 | CACNA1H | 123 | CHUK |
| 28 | FKBP1A | 60 | PIK3R1 | 92 | DCUN1D1 |  |  |
| 29 | HDAC2 | 61 | PSMB1 | 93 | MMP8 |  |  |
| 30 | PRKCD | 62 | GLRA1 | 94 | HTR7 |  |  |
| 31 | TFPI | 63 | STING1 | 95 | S1PR5 |  |  |
| 32 | F13A1 | 64 | PDE3A | 96 | LNPEP |  |  |

**Table S5.** Main targets of Alisol B monoacetate

| No. | target | No. | target | No. | target | No. | target |
| --- | --- | --- | --- | --- | --- | --- | --- |
| 1 | APEX1 | 34 | PIK3R1 | 67 | PTGER2 | 100 | DCUN1D1 |
| 2 | NFKB1 | 35 | TOP2A | 68 | PDPK1 | 101 | NR4A1 |
| 3 | CTSD | 36 | PIK3CB | 69 | QRFPR | 102 | CYSLTR2 |
| 4 | GPR55 | 37 | SLC6A3 | 70 | F7 | 103 | BLM |
| 5 | F2 | 38 | STAT3 | 71 | RPS6KA1 | 104 | AHCY |
| 6 | CTSL | 39 | MC4R | 72 | PIK3CD | 105 | THRA |
| 7 | FPR1 | 40 | S1PR5 | 73 | GBA1 | 106 | PDGFRA |
| 8 | CLK4 | 41 | BCHE | 74 | OPRD1 | 107 | STK3 |
| 9 | KDM1A | 42 | CCR1 | 75 | ABCC1 | 108 | PDE3A |
| 10 | GLRA1 | 43 | SCN3A | 76 | APLNR | 109 | NT5E |
| 11 | ACACA | 44 | CACNA1B | 77 | PSMB9 | 110 | CTSB |
| 12 | TDP1 | 45 | TFPI | 78 | NR1I2 | 111 | OPRK1 |
| 13 | NR3C2 | 46 | MTOR | 79 | CDK5 | 112 | FER |
| 14 | PTGS1 | 47 | CNR2 | 80 | PTPN2 | 113 | MAP3K14 |
| 15 | DUSP3 | 48 | CSNK2B | 81 | BCL6 | 114 | SERPINE1 |
| 16 | CYP3A4 | 49 | PRCP | 82 | NTSR2 | 115 | CCR2 |
| 17 | FPR2 | 50 | CDK1 | 83 | PTGER1 | 116 | CLK1 |
| 18 | HDAC10 | 51 | SCD | 84 | ULK3 | 117 | TNK1 |
| 19 | AKR1C3 | 52 | SCN2A | 85 | P2RX7 | 118 | SLC9A1 |
| 20 | C5AR1 | 53 | RORB | 86 | PROC | 119 | EP300 |
| 21 | TRIM24 | 54 | P2RY12 | 87 | MMP7 | 120 | ST14 |
| 22 | HDAC2 | 55 | CDC25C | 88 | CHUK | 121 | P2RY2 |
| 23 | TMPRSS6 | 56 | ADRB1 | 89 | CACNA1H | 122 | ANPEP |
| 24 | AURKB | 57 | PLG | 90 | PIK3CA | 123 | PRKCD |
| 25 | IDO1 | 58 | RXFP1 | 91 | ZAP70 | 124 | MCHR1 |
| 26 | HIF1A | 59 | ITK | 92 | ACACB | 125 | NAMPT |
| 27 | KLF5 | 60 | NOTUM | 93 | ADORA1 | 126 | HSD17B10 |
| 28 | CFTR | 61 | PIK3CG | 94 | AR | 127 | CASP6 |
| 29 | SLC6A5 | 62 | ITGB1 | 95 | METAP2 | 128 | TDP2 |
| 30 | ADAM10 | 63 | TLR4 | 96 | FKBP1A | 129 | RPS6KA6 |
| 31 | GRIN1 | 64 | ITGB3 | 97 | HDAC4 | 130 | LNPEP |
| 32 | FBP1 | 65 | CCNE1 | 98 | AOC3 |  |  |
| 33 | NTRK3 | 66 | WDR5 | 99 | SPHK1 |  |  |

**Table S6.** Main targets of alisol C monoacetate

| No. | target | No. | target | No. | target | No. | target |
| --- | --- | --- | --- | --- | --- | --- | --- |
| 1 | APEX1 | 36 | PIK3R1 | 71 | RORB | 106 | LNPEP |
| 2 | NFKB1 | 37 | SCN3A | 72 | PTPN2 | 107 | SPHK1 |
| 3 | CTSD | 38 | KLF5 | 73 | ACACB | 108 | AOC3 |
| 4 | CLK4 | 39 | MTOR | 74 | ITGB3 | 109 | CHUK |
| 5 | ACACA | 40 | CA1 | 75 | PLG | 110 | ABCB1 |
| 6 | CTSL | 41 | SLC6A3 | 76 | PROC | 111 | CXCR4 |
| 7 | FPR1 | 42 | CSNK2B | 77 | RPS6KA1 | 112 | ABCC1 |
| 8 | MC4R | 43 | TOP2A | 78 | WDR5 | 113 | ANPEP |
| 9 | F2 | 44 | SLC6A5 | 79 | QRFPR | 114 | PTGER1 |
| 10 | KDM1A | 45 | NTRK3 | 80 | PTGER2 | 115 | ADAM17 |
| 11 | STAT3 | 46 | SCN2A | 81 | PDPK1 | 116 | P2RY2 |
| 12 | TDP1 | 47 | PIK3CG | 82 | HDAC1 | 117 | SAE1 |
| 13 | GPR55 | 48 | ITK | 83 | ADRB1 | 118 | FCGRT |
| 14 | HDAC2 | 49 | TFPI | 84 | CLK1 | 119 | SLC9A1 |
| 15 | PIK3CB | 50 | S1PR5 | 85 | DCUN1D1 | 120 | CYSLTR2 |
| 16 | DUSP3 | 51 | RXFP1 | 86 | BCL6 | 121 | MMP7 |
| 17 | AURKB | 52 | IDO1 | 87 | PRKCD | 122 | AR |
| 18 | FBP1 | 53 | FKBP1A | 88 | AKR1C3 | 123 | NR1I2 |
| 19 | GLRA1 | 54 | PRCP | 89 | NTSR2 | 124 | HCAR2 |
| 20 | HDAC10 | 55 | PIK3CA | 90 | CCNE1 | 125 | MAP3K14 |
| 21 | CNR2 | 56 | CDK1 | 91 | OPRK1 | 126 | TYRO3 |
| 22 | NR3C2 | 57 | HSP90AB1 | 92 | P2RX7 | 127 | DRD3 |
| 23 | FPR2 | 58 | NOTUM | 93 | ZAP70 | 128 | PSMB1 |
| 24 | CCR1 | 59 | ADORA1 | 94 | PDGFRA | 129 | AKT3 |
| 25 | CYP3A4 | 60 | HDAC4 | 95 | APLNR | 130 | RORC |
| 26 | TRIM24 | 61 | F7 | 96 | CACNA1H | 131 | DPP7 |
| 27 | ADAM10 | 62 | CDK5 | 97 | PRKCA | 132 | TNK1 |
| 28 | C5AR1 | 63 | PDE3A | 98 | METAP2 | 133 | LTA4H |
| 29 | OPRD1 | 64 | SCD | 99 | MAP2K2 | 134 | FER |
| 30 | PTGS1 | 65 | CDC25C | 100 | SERPINE1 | 135 | HDAC11 |
| 31 | TMPRSS6 | 66 | P2RY12 | 101 | RPS6KA6 | 136 | ST14 |
| 32 | CFTR | 67 | PSMB9 | 102 | THRA | 137 | CTSB |
| 33 | HIF1A | 68 | TLR4 | 103 | CASP6 | 138 | CCR2 |
| 34 | CACNA1B | 69 | ITGB1 | 104 | NR4A1 |  |  |
| 35 | GRIN1 | 70 | PIK3CD | 105 | NT5E |  |  |

**Table S7.** Main targets of beta-sitosterol

| No. | target | No. | target | No. | target | No. | target |
| --- | --- | --- | --- | --- | --- | --- | --- |
| 1 | NFKB1 | 32 | VDR | 63 | CAMK2D | 94 | EZH2 |
| 2 | CTSD | 33 | C5AR1 | 64 | CCR1 | 95 | IDO1 |
| 3 | ADORA1 | 34 | TMPRSS6 | 65 | CYSLTR2 | 96 | PIK3R1 |
| 4 | CNR2 | 35 | ABCC1 | 66 | METAP2 | 97 | MMP8 |
| 5 | CLK4 | 36 | CCNE1 | 67 | SLC9A1 | 98 | P2RX7 |
| 6 | PTGS1 | 37 | ACACA | 68 | TDO2 | 99 | LDHA |
| 7 | NR3C2 | 38 | CACNA1B | 69 | CDC25C | 100 | CDK2 |
| 8 | GRIN1 | 39 | SLC6A3 | 70 | PDE3B | 101 | HSD17B10 |
| 9 | GPR55 | 40 | PSMB1 | 71 | DPP9 | 102 | SLC2A1 |
| 10 | FPR1 | 41 | KIF11 | 72 | CHRM4 | 103 | CDK5 |
| 11 | GLRA1 | 42 | SCN3A | 73 | PIK3CD | 104 | ERAP1 |
| 12 | KDM1A | 43 | NTSR2 | 74 | SCN2A | 105 | MMP1 |
| 13 | APEX1 | 44 | ANPEP | 75 | RPS6KA1 | 106 | TLR4 |
| 14 | NTRK3 | 45 | PTPN2 | 76 | NOS3 | 107 | HTR2A |
| 15 | AR | 46 | DHFR | 77 | SCD | 108 | PSMB5 |
| 16 | FPR2 | 47 | PROC | 78 | CLK1 | 109 | RXFP1 |
| 17 | HTR2C | 48 | SLC6A5 | 79 | PRKCA | 110 | SLC40A1 |
| 18 | KLF5 | 49 | DCUN1D1 | 80 | CTSB | 111 | PIK3CB |
| 19 | PSMB2 | 50 | CHRNB4 | 81 | NR1I2 | 112 | PSMB9 |
| 20 | AKR1C3 | 51 | S1PR5 | 82 | ACACB | 113 | BMP2K |
| 21 | KCNH2 | 52 | PRCP | 83 | AAK1 | 114 | PAK4 |
| 22 | HDAC8 | 53 | CFTR | 84 | CTSL | 115 | CBX4 |
| 23 | ITK | 54 | OPRK1 | 85 | CHRM5 | 116 | GSTP1 |
| 24 | ESR2 | 55 | ADORA2B | 86 | GBA1 | 117 | NTRK1 |
| 25 | STAT3 | 56 | CACNA1H | 87 | HDAC10 | 118 | S1PR2 |
| 26 | TRIM24 | 57 | NFE2L2 | 88 | CTSK | 119 | P2RY6 |
| 27 | TOP2A | 58 | HSD11B2 | 89 | MARK3 | 120 | QRFPR |
| 28 | MTOR | 59 | APH1A | 90 | CDK7 | 121 | CASP8 |
| 29 | PRKCD | 60 | HDAC2 | 91 | SPHK1 |  |  |
| 30 | AURKB | 61 | CLK2 | 92 | GHSR |  |  |
| 31 | ADAM10 | 62 | HTR7 | 93 | PDGFRB |  |  |

**Table S8.** Main targets of hederagenin

| No. | target | No. | target | No. | target | No. | target |
| --- | --- | --- | --- | --- | --- | --- | --- |
| 1 | BLM | 24 | PDGFRA | 47 | HDAC7 | 70 | NR4A1 |
| 2 | GPR55 | 25 | DPP9 | 48 | S1PR5 | 71 | PRCP |
| 3 | PTPN1 | 26 | GLRA1 | 49 | PSMB9 | 72 | NR2E3 |
| 4 | KDM1A | 27 | CYP3A4 | 50 | IDO1 | 73 | SOAT1 |
| 5 | PTGS1 | 28 | KLF5 | 51 | AKR1C3 | 74 | CFTR |
| 6 | CLK4 | 29 | NTSR2 | 52 | CHRM4 | 75 | CNR2 |
| 7 | NFKB1 | 30 | SLC6A5 | 53 | SLC1A3 | 76 | GPR6 |
| 8 | TRIM24 | 31 | CSNK2B | 54 | DPP8 | 77 | P2RX7 |
| 9 | APEX1 | 32 | TLR4 | 55 | HDAC11 | 78 | PROC |
| 10 | NR3C2 | 33 | GRIN1 | 56 | NTRK1 | 79 | CDC25C |
| 11 | CDC25B | 34 | TACR2 | 57 | F11 | 80 | PTK2B |
| 12 | PTPN2 | 35 | CHRM1 | 58 | CACNA1B | 81 | ANPEP |
| 13 | NTRK3 | 36 | ITK | 59 | CACNA1H | 82 | MMP1 |
| 14 | ADAM10 | 37 | CDK5 | 60 | ACACA | 83 | RORB |
| 15 | PIK3R1 | 38 | C5AR1 | 61 | ACACB | 84 | RPS6KA1 |
| 16 | TERT | 39 | ATG4B | 62 | PDE3A | 85 | PRKACA |
| 17 | CTSD | 40 | TLR8 | 63 | WDR5 | 86 | S1PR2 |
| 18 | HSP90AB1 | 41 | CYSLTR2 | 64 | ADORA1 | 87 | ABCB1 |
| 19 | SCN2A | 42 | PSMB1 | 65 | CHRM5 | 88 | GRIA2 |
| 20 | STAT3 | 43 | AURKB | 66 | SLC40A1 | 89 | KIF11 |
| 21 | TOP2A | 44 | SLC9A1 | 67 | F13A1 |  |  |
| 22 | SCN3A | 45 | AR | 68 | CBX4 |  |  |
| 23 | FPR2 | 46 | MTOR | 69 | FPR1 |  |  |

**Table S9.** Main targets of Kaempferol

| No. | target | No. | target | No. | target | No. | target |
| --- | --- | --- | --- | --- | --- | --- | --- |
| 1 | HSD17B10 | 32 | PDE11A | 63 | STK3 | 94 | GLS |
| 2 | APEX1 | 33 | STAT1 | 64 | GSK3B | 95 | KDM4A |
| 3 | TDP1 | 34 | HDAC8 | 65 | MAPKAPK2 | 96 | TFPI |
| 4 | RPS6KA3 | 35 | AKR1C1 | 66 | ACACA | 97 | CDK1 |
| 5 | TOP2A | 36 | CDK2 | 67 | NR3C2 | 98 | NFKB1 |
| 6 | XDH | 37 | PIN1 | 68 | QRFPR | 99 | SCN3A |
| 7 | CTSD | 38 | PSMB1 | 69 | CDC25C | 100 | SLC2A1 |
| 8 | TTR | 39 | ACVR1B | 70 | ERAP1 | 101 | MC5R |
| 9 | MAOA | 40 | CXCR4 | 71 | PIK3R1 | 102 | MIF |
| 10 | ESR2 | 41 | FCGRT | 72 | PDE3A | 103 | ULK3 |
| 11 | ALOX12 | 42 | CDK5 | 73 | TNIK | 104 | SLC1A2 |
| 12 | DUSP3 | 43 | LGALS3 | 74 | NPC1 | 105 | DCUN1D1 |
| 13 | THRA | 44 | NR4A1 | 75 | NLK | 106 | SLC1A1 |
| 14 | NR1I2 | 45 | RORB | 76 | AKR1C2 | 107 | S1PR4 |
| 15 | KLF5 | 46 | BMP2K | 77 | HPGD | 108 | IMPDH2 |
| 16 | TRIM24 | 47 | GRIA2 | 78 | MTOR | 109 | GRIN1 |
| 17 | CLK4 | 48 | GRK5 | 79 | P2RX4 | 110 | SCD |
| 18 | GLRA1 | 49 | CDC25B | 80 | TBXA2R | 111 | MME |
| 19 | CSNK2B | 50 | PRCP | 81 | NTSR2 | 112 | SERPINE1 |
| 20 | RPS6KA1 | 51 | LDHB | 82 | PRKCZ | 113 | CACNA1B |
| 21 | AAK1 | 52 | NTRK3 | 83 | GUSB | 114 | TYRO3 |
| 22 | YES1 | 53 | NQO2 | 84 | GPR17 | 115 | PLAT |
| 23 | GPBAR1 | 54 | AVPR1B | 85 | CHKA | 116 | DRD2 |
| 24 | FFAR4 | 55 | HDAC2 | 86 | PTGER1 | 117 | PDE3B |
| 25 | TBK1 | 56 | METAP2 | 87 | MAP2K2 | 118 | CHRM5 |
| 26 | F13A1 | 57 | MELK | 88 | ZAP70 | 119 | ADRB1 |
| 27 | SLC6A5 | 58 | CFTR | 89 | NOS2 | 120 | AKR1B1 |
| 28 | RPS6KA6 | 59 | GBA2 | 90 | CBX4 | 121 | MAPK3 |
| 29 | ERN1 | 60 | CAPN1 | 91 | MAP3K11 |  |  |
| 30 | PTPN11 | 61 | EGLN1 | 92 | CHRNB4 |  |  |
| 31 | GLO1 | 62 | CAMKK2 | 93 | MINK1 |  |  |

**Table S10.** Main targets of Mandenol

| No. | target | No. | target | No. | target | No. | target |
| --- | --- | --- | --- | --- | --- | --- | --- |
| 1 | PTGS2 | 28 | CSNK2B | 55 | KCNH2 | 82 | KAT6A |
| 2 | SLC6A5 | 29 | NR4A1 | 56 | AURKB | 83 | NAMPT |
| 3 | NFKB1 | 30 | CHRNB4 | 57 | DCTPP1 | 84 | GPR17 |
| 4 | CES1 | 31 | SCN4A | 58 | ABCC1 | 85 | RPS6KA5 |
| 5 | GGPS1 | 32 | TOP2A | 59 | FGR | 86 | PDE3B |
| 6 | CTSD | 33 | RORB | 60 | CYSLTR2 | 87 | METAP2 |
| 7 | DPP8 | 34 | SCN3A | 61 | PIK3CA | 88 | GPBAR1 |
| 8 | DPP9 | 35 | CYP3A4 | 62 | PRKCD | 89 | DCUN1D1 |
| 9 | TDP1 | 36 | CDK1 | 63 | P2RY12 | 90 | GRK5 |
| 10 | S1PR3 | 37 | PIN1 | 64 | KCNK9 | 91 | AOC3 |
| 11 | KLF5 | 38 | NOS2 | 65 | PIK3CB | 92 | PTGER1 |
| 12 | NFE2L2 | 39 | CLK4 | 66 | CYP2A6 | 93 | NR3C2 |
| 13 | TLR4 | 40 | RXFP1 | 67 | GUSB | 94 | KEAP1 |
| 14 | NR1I2 | 41 | PIK3CD | 68 | PDGFRB | 95 | PLA2G2A |
| 15 | TOP1 | 42 | CAPN1 | 69 | CPT2 | 96 | SLC9A1 |
| 16 | PTGS1 | 43 | GLRA1 | 70 | PTPN2 | 97 | PDGFRA |
| 17 | PIK3R1 | 44 | NR2E3 | 71 | PSMB9 | 98 | TAOK3 |
| 18 | S1PR5 | 45 | FLT1 | 72 | SLC40A1 | 99 | PIK3CG |
| 19 | ERAP2 | 46 | ADAM10 | 73 | NTRK3 | 100 | SLC2A1 |
| 20 | AHCY | 47 | PRKCA | 74 | NTSR2 | 101 | STAT1 |
| 21 | FPR1 | 48 | ANPEP | 75 | HSD17B10 | 102 | KLK7 |
| 22 | S1PR2 | 49 | APEX1 | 76 | ALOX5 | 103 | KIF11 |
| 23 | TRIM24 | 50 | CDK5 | 77 | MAP2K2 | 104 | CACNA1C |
| 24 | FPR2 | 51 | EPHX1 | 78 | C5AR1 | 105 | PSMB1 |
| 25 | GPR35 | 52 | NQO2 | 79 | PTPN1 | 106 | CES2 |
| 26 | PPARA | 53 | HTR2C | 80 | SCN2A | 107 | KDR |
| 27 | CA3 | 54 | GRIN1 | 81 | KDM1A |  |  |

**Table S11.** Main targets of Myricanone

| No. | target | No. | target | No. | target | No. | target |
| --- | --- | --- | --- | --- | --- | --- | --- |
| 1 | APEX1 | 28 | PDGFRA | 55 | NR3C2 | 82 | NR1I2 |
| 2 | TRIM24 | 29 | CDC25C | 56 | RET | 83 | QRFPR |
| 3 | CTSD | 30 | EGLN1 | 57 | F13A1 | 84 | ANPEP |
| 4 | ALOX12 | 31 | CDC25B | 58 | PIK3R1 | 85 | CHRM4 |
| 5 | HSD17B10 | 32 | ERN1 | 59 | CCNE1 | 86 | MIF |
| 6 | MAOA | 33 | GCK | 60 | ENPP1 | 87 | NAMPT |
| 7 | NFKB1 | 34 | GPBAR1 | 61 | TNIK | 88 | TYRO3 |
| 8 | HDAC8 | 35 | TTR | 62 | PTK2B | 89 | MME |
| 9 | KLF5 | 36 | TDP1 | 63 | AVPR1B | 90 | IMPDH2 |
| 10 | DUSP3 | 37 | CSNK2B | 64 | CHUK | 91 | CXCR4 |
| 11 | SLC2A1 | 38 | PLA2G2A | 65 | CYP3A4 | 92 | SLC9A1 |
| 12 | PSMB1 | 39 | LDHB | 66 | GHSR | 93 | HTR1A |
| 13 | THRA | 40 | KEAP1 | 67 | GPR55 | 94 | FKBP1A |
| 14 | RORB | 41 | TFPI | 68 | ACACA | 95 | FGR |
| 15 | NTRK3 | 42 | CDK5 | 69 | PTPN11 | 96 | PDGFRB |
| 16 | DRD1 | 43 | GLS | 70 | PIN1 | 97 | METAP2 |
| 17 | PDE3A | 44 | S1PR5 | 71 | NFE2L2 | 98 | DCUN1D1 |
| 18 | TOP2A | 45 | KDM1A | 72 | GABRA1 | 99 | EIF2AK1 |
| 19 | CDK1 | 46 | SCD | 73 | SAE1 | 100 | ACACB |
| 20 | IDO1 | 47 | PSMB2 | 74 | ITGB1 | 101 | PRKCZ |
| 21 | SLC6A5 | 48 | CFTR | 75 | MAP3K11 | 102 | NOX1 |
| 22 | GLRA1 | 49 | KCNA5 | 76 | ALOX5 | 103 | HIF1A |
| 23 | HDAC2 | 50 | DRD2 | 77 | DPP9 | 104 | ALK |
| 24 | XDH | 51 | CCR1 | 78 | PLAT | 105 | SLC1A3 |
| 25 | CLK4 | 52 | FPR1 | 79 | ADAM10 |  |  |
| 26 | ACVRL1 | 53 | AXL | 80 | GUSB |  |  |
| 27 | C5AR1 | 54 | ADRB1 | 81 | MDM4 |  |  |

**Table S12.** Main targets of Paeoniflorgenone

| No. | target | No. | target | No. | target | No. | target |
| --- | --- | --- | --- | --- | --- | --- | --- |
| 1 | APEX1 | 21 | ACACA | 41 | SAE1 | 61 | MARK4 |
| 2 | NFKB1 | 22 | CDK5 | 42 | SCN3A | 62 | STK3 |
| 3 | KLF5 | 23 | TOP2A | 43 | NR3C2 | 63 | AURKB |
| 4 | PTGS1 | 24 | NTRK3 | 44 | CLK4 | 64 | SCN4A |
| 5 | KDM1A | 25 | NR1I2 | 45 | SLC2A1 | 65 | RPS6KA1 |
| 6 | CTSD | 26 | CHRM1 | 46 | FPR1 | 66 | GRB2 |
| 7 | TRIM24 | 27 | SLC6A5 | 47 | ITK | 67 | GRK5 |
| 8 | CSNK2B | 28 | CNR2 | 48 | HRH3 | 68 | CFTR |
| 9 | KCNH2 | 29 | DUSP3 | 49 | CHUK | 69 | TDP1 |
| 10 | SERPINE1 | 30 | RORB | 50 | HDAC4 | 70 | MELK |
| 11 | CHRM5 | 31 | C5AR1 | 51 | TACR2 | 71 | ITGB1 |
| 12 | ADAM10 | 32 | NFE2L2 | 52 | HDAC5 | 72 | DPP9 |
| 13 | PSMB1 | 33 | TRPA1 | 53 | GRIN1 | 73 | CYSLTR2 |
| 14 | CYP3A4 | 34 | PTPN2 | 54 | PRKCD | 74 | NTSR2 |
| 15 | MAOA | 35 | CAPN1 | 55 | PSMB9 | 75 | STING1 |
| 16 | CACNA1B | 36 | PRCP | 56 | TLR4 | 76 | S1PR5 |
| 17 | PDGFRA | 37 | CDC25C | 57 | TFPI | 77 | TGFBR2 |
| 18 | CHRM4 | 38 | PRSS1 | 58 | CNR1 | 78 | HSP90AB1 |
| 19 | MAP2K2 | 39 | FPR2 | 59 | NOS2 | 79 | GBA1 |
| 20 | HIF1A | 40 | GABRA1 | 60 | ADORA1 | 80 | F13A1 |

**Table S13.** Main targets of Stigmasterol

| No. | target | No. | target | No. | target | No. | target |
| --- | --- | --- | --- | --- | --- | --- | --- |
| 1 | NFKB1 | 36 | ACACA | 71 | CCR1 | 106 | PSMB5 |
| 2 | ADORA1 | 37 | TMPRSS6 | 72 | IDO1 | 107 | APH1A |
| 3 | CNR2 | 38 | S1PR5 | 73 | MMP2 | 108 | P2RY12 |
| 4 | CTSD | 39 | AKR1C3 | 74 | SLC40A1 | 109 | SLC2A1 |
| 5 | PTGS1 | 40 | PRKCA | 75 | RXFP1 | 110 | CNR1 |
| 6 | TDP1 | 41 | PSMB2 | 76 | SCN3A | 111 | NFE2L2 |
| 7 | CLK4 | 42 | PRKCD | 77 | SLC9A1 | 112 | MMP7 |
| 8 | NR3C2 | 43 | CDK7 | 78 | CCNE1 | 113 | PDGFRA |
| 9 | APEX1 | 44 | CLK1 | 79 | PTPN2 | 114 | PDE7A |
| 10 | GRIN1 | 45 | SLC6A5 | 80 | CHRNB4 | 115 | PIK3R1 |
| 11 | FPR1 | 46 | CACNA1B | 81 | TLR4 | 116 | FBP1 |
| 12 | KDM1A | 47 | PIK3CB | 82 | SCD | 117 | CTSL |
| 13 | GLRA1 | 48 | PIK3CA | 83 | PSMB1 | 118 | ESR1 |
| 14 | FPR2 | 49 | ADORA2B | 84 | PDGFRB | 119 | F11 |
| 15 | ESR2 | 50 | GBA1 | 85 | CACNA1H | 120 | SPHK1 |
| 16 | OPRD1 | 51 | AAK1 | 86 | ANPEP | 121 | IDH1 |
| 17 | GPR55 | 52 | PROC | 87 | HDAC10 | 122 | CAMK2D |
| 18 | NTRK3 | 53 | NTSR2 | 88 | ACACB | 123 | TERT |
| 19 | AR | 54 | HDAC2 | 89 | NR1I2 | 124 | CDK5 |
| 20 | TRIM24 | 55 | TDO2 | 90 | RPS6KA1 | 125 | BLM |
| 21 | ITK | 56 | HTR2C | 91 | HSD11B2 | 126 | HDAC5 |
| 22 | AURKB | 57 | MTOR | 92 | PDE3B | 127 | GCK |
| 23 | DHFR | 58 | C5AR1 | 93 | F7 | 128 | PRKCB |
| 24 | ADAM10 | 59 | PTK2B | 94 | EZH2 | 129 | TFPI |
| 25 | CFTR | 60 | S1PR3 | 95 | FKBP1A | 130 | NR4A1 |
| 26 | TOP2A | 61 | BMP2K | 96 | CA3 | 131 | PDPK1 |
| 27 | KLF5 | 62 | SLC6A3 | 97 | HTR7 | 132 | CHUK |
| 28 | OPRK1 | 63 | DCUN1D1 | 98 | CLK2 | 133 | TAOK1 |
| 29 | CTSB | 64 | PRCP | 99 | CYSLTR2 | 134 | CASP8 |
| 30 | S1PR2 | 65 | CTSK | 100 | RORC | 135 | TYRO3 |
| 31 | VDR | 66 | MMP8 | 101 | MMP1 | 136 | LDHA |
| 32 | DPP9 | 67 | HSP90AB1 | 102 | UGT2B7 | 137 | WDR5 |
| 33 | MARK3 | 68 | KIF11 | 103 | METAP2 | 138 | SCN4A |
| 34 | ABCC1 | 69 | PIK3CD | 104 | DUT |  |  |
| 35 | STAT3 | 70 | SCN2A | 105 | CCR2 |  |  |

**Table S14.** Main targets of Wallichilide

| No. | target | No. | target | No. | target | No. | target |
| --- | --- | --- | --- | --- | --- | --- | --- |
| 1 | NFE2L2 | 25 | PDGFRB | 49 | CSNK2B | 73 | CLK4 |
| 2 | TDP1 | 26 | PIK3R1 | 50 | SLC6A5 | 74 | PSMB1 |
| 3 | GPR17 | 27 | CNR1 | 51 | RORB | 75 | FPR2 |
| 4 | WDR5 | 28 | PTGS1 | 52 | TRIM24 | 76 | HSP90AB1 |
| 5 | GPR55 | 29 | S1PR5 | 53 | KDM1A | 77 | CYSLTR2 |
| 6 | KLF5 | 30 | PTGS2 | 54 | CBX4 | 78 | NR2E3 |
| 7 | GPR35 | 31 | KCNK9 | 55 | NFKB1 | 79 | CACNA1B |
| 8 | RXFP1 | 32 | C5AR1 | 56 | PIK3CG | 80 | F13A1 |
| 9 | TMPRSS6 | 33 | KCNH2 | 57 | PTPN1 | 81 | MMP8 |
| 10 | TOP2A | 34 | PDE3A | 58 | ACACA | 82 | DPP8 |
| 11 | AR | 35 | CCR1 | 59 | FPR1 | 83 | GGPS1 |
| 12 | CDK5 | 36 | NTSR2 | 60 | CYP3A4 | 84 | DPP9 |
| 13 | CDK1 | 37 | CNR2 | 61 | NR1I2 | 85 | ADAM10 |
| 14 | GRIN1 | 38 | SLC2A1 | 62 | AURKB | 86 | SCN3A |
| 15 | CCNE1 | 39 | ADORA2B | 63 | SERPINE1 | 87 | TLR4 |
| 16 | PSMB9 | 40 | CTSD | 64 | CHUK | 88 | SCD |
| 17 | EPHX1 | 41 | DUSP3 | 65 | ALOX12 | 89 | NTRK3 |
| 18 | NR3C2 | 42 | AHCY | 66 | CASP8 | 90 | APEX1 |
| 19 | P2RY12 | 43 | SLC9A1 | 67 | PTPN2 | 91 | GLRA1 |
| 20 | PDGFRA | 44 | GSK3A | 68 | CAPN1 | 92 | QRFPR |
| 21 | NR3C1 | 45 | S1PR2 | 69 | METAP2 | 93 | NT5E |
| 22 | KEAP1 | 46 | ITK | 70 | STAT3 | 94 | STAT1 |
| 23 | PRSS1 | 47 | PRKCA | 71 | CFTR |  |  |
| 24 | GABRA1 | 48 | ABCC1 | 72 | SCN2A |  |  |

**Table S15.** Potential targets of Danggui Shaoyao San (DSS) and Depression.

| **No.** | **target** | **No.** | **target** | **No.** | **target** | **No.** | **target** |
| --- | --- | --- | --- | --- | --- | --- | --- |
| 1 | AAK1 | 87 | DPP7 | 173 | KCNK9 | 259 | PRKCD |
| 2 | ABCB1 | 88 | DPP8 | 174 | KDM1A | 260 | PRKCQ |
| 3 | ABCC1 | 89 | DPP9 | 175 | KDM4A | 261 | PRKCZ |
| 4 | ACACA | 90 | DRD1 | 176 | KDR | 262 | PROC |
| 5 | ACACB | 91 | DRD2 | 177 | KEAP1 | 263 | PRSS1 |
| 6 | ACVR1B | 92 | DRD3 | 178 | KIF11 | 264 | PSMB1 |
| 7 | ACVRL1 | 93 | DUSP3 | 179 | KLF5 | 265 | PSMB2 |
| 8 | ADAM10 | 94 | DUT | 180 | KLK7 | 266 | PSMB5 |
| 9 | ADAM17 | 95 | EGLN1 | 181 | LDHA | 267 | PSMB9 |
| 10 | ADORA1 | 96 | EIF2AK1 | 182 | LDHB | 268 | PTGER1 |
| 11 | ADORA2B | 97 | ENPP1 | 183 | LGALS3 | 269 | PTGER2 |
| 12 | ADRB1 | 98 | EP300 | 184 | LNPEP | 270 | PTGS1 |
| 13 | AHCY | 99 | EPHB2 | 185 | LTA4H | 271 | PTGS2 |
| 14 | AKR1B1 | 100 | EPHX1 | 186 | MAOA | 272 | PTK2B |
| 15 | AKR1C1 | 101 | ERAP1 | 187 | MAP2K2 | 273 | PTPN1 |
| 16 | AKR1C2 | 102 | ERAP2 | 188 | MAP3K11 | 274 | PTPN11 |
| 17 | AKR1C3 | 103 | ERN1 | 189 | MAP3K14 | 275 | PTPN2 |
| 18 | AKT3 | 104 | ESR1 | 190 | MAP3K5 | 276 | QRFPR |
| 19 | ALK | 105 | ESR2 | 191 | MAPK3 | 277 | RAF1 |
| 20 | ALOX12 | 106 | EZH2 | 192 | MAPKAPK2 | 278 | RET |
| 21 | ALOX5 | 107 | F11 | 193 | MARK3 | 279 | ROCK1 |
| 22 | ANPEP | 108 | F13A1 | 194 | MARK4 | 280 | RORB |
| 23 | AOC3 | 109 | F2 | 195 | MC4R | 281 | RORC |
| 24 | APEX1 | 110 | F7 | 196 | MC5R | 282 | RPS6KA1 |
| 25 | APH1A | 111 | FBP1 | 197 | MCHR1 | 283 | RPS6KA3 |
| 26 | APLNR | 112 | FCGRT | 198 | MDM4 | 284 | RPS6KA5 |
| 27 | AR | 113 | FER | 199 | MELK | 285 | RPS6KA6 |
| 28 | ATG4B | 114 | FFAR4 | 200 | METAP2 | 286 | RXFP1 |
| 29 | AURKB | 115 | FGR | 201 | MIF | 287 | RXRB |
| 30 | AVPR1B | 116 | FKBP1A | 202 | MINK1 | 288 | S1PR2 |
| 31 | AXL | 117 | FLT1 | 203 | MME | 289 | S1PR3 |
| 32 | BCHE | 118 | FPR1 | 204 | MMP1 | 290 | S1PR4 |
| 33 | BCL6 | 119 | FPR2 | 205 | MMP2 | 291 | S1PR5 |
| 34 | BLM | 120 | GABRA1 | 206 | MMP7 | 292 | SAE1 |
| 35 | BMP2K | 121 | GBA1 | 207 | MMP8 | 293 | SCD |
| 36 | BRDT | 122 | GBA2 | 208 | MMP9 | 294 | SCN2A |
| 37 | C5AR1 | 123 | GCK | 209 | MTOR | 295 | SCN3A |
| 38 | CA1 | 124 | GGPS1 | 210 | NAMPT | 296 | SCN4A |
| 39 | CA3 | 125 | GHSR | 211 | NFE2L2 | 297 | SERPINE1 |
| 40 | CACNA1B | 126 | GLO1 | 212 | NFKB1 | 298 | SLC1A1 |
| 41 | CACNA1C | 127 | GLRA1 | 213 | NLK | 299 | SLC1A2 |
| 42 | CACNA1H | 128 | GLS | 214 | NOS2 | 300 | SLC1A3 |
| 43 | CAMK2D | 129 | GPBAR1 | 215 | NOS3 | 301 | SLC2A1 |
| 44 | CAMKK2 | 130 | GPR17 | 216 | NOTUM | 302 | SLC40A1 |
| 45 | CAPN1 | 131 | GPR35 | 217 | NOX1 | 303 | SLC6A3 |
| 46 | CASP6 | 132 | GPR55 | 218 | NPC1 | 304 | SLC6A4 |
| 47 | CASP8 | 133 | GPR6 | 219 | NQO2 | 305 | SLC6A5 |
| 48 | CBX4 | 134 | GRB2 | 220 | NR1I2 | 306 | SLC9A1 |
| 49 | CCNE1 | 135 | GRIA2 | 221 | NR2E3 | 307 | SOAT1 |
| 50 | CCR1 | 136 | GRIN1 | 222 | NR3C1 | 308 | SPHK1 |
| 51 | CCR2 | 137 | GRK5 | 223 | NR3C2 | 309 | ST14 |
| 52 | CDC25B | 138 | GSK3A | 224 | NR4A1 | 310 | STAT1 |
| 53 | CDC25C | 139 | GSK3B | 225 | NT5E | 311 | STAT3 |
| 54 | CDK1 | 140 | GSTP1 | 226 | NTRK1 | 312 | STING1 |
| 55 | CDK2 | 141 | GUSB | 227 | NTRK3 | 313 | STK3 |
| 56 | CDK4 | 142 | HCAR2 | 228 | NTSR2 | 314 | TACR2 |
| 57 | CDK5 | 143 | HDAC1 | 229 | OPRD1 | 315 | TAOK1 |
| 58 | CDK7 | 144 | HDAC10 | 230 | OPRK1 | 316 | TAOK3 |
| 59 | CES1 | 145 | HDAC11 | 231 | OPRM1 | 317 | TBK1 |
| 60 | CES2 | 146 | HDAC2 | 232 | P2RX4 | 318 | TBXA2R |
| 61 | CFTR | 147 | HDAC4 | 233 | P2RX7 | 319 | TDO2 |
| 62 | CHKA | 148 | HDAC5 | 234 | P2RY12 | 320 | TDP1 |
| 63 | CHRM1 | 149 | HDAC7 | 235 | P2RY2 | 321 | TDP2 |
| 64 | CHRM4 | 150 | HDAC8 | 236 | P2RY6 | 322 | TERT |
| 65 | CHRM5 | 151 | HDAC9 | 237 | PAK4 | 323 | TFPI |
| 66 | CHRNB4 | 152 | HIF1A | 238 | PDE11A | 324 | TGFBR2 |
| 67 | CHUK | 153 | HPGD | 239 | PDE3A | 325 | THRA |
| 68 | CLK1 | 154 | HRH3 | 240 | PDE3B | 326 | TLR4 |
| 69 | CLK2 | 155 | HSD11B2 | 241 | PDE7A | 327 | TLR8 |
| 70 | CLK4 | 156 | HSD17B10 | 242 | PDGFRA | 328 | TMPRSS6 |
| 71 | CNR1 | 157 | HSP90AA1 | 243 | PDGFRB | 329 | TNIK |
| 72 | CNR2 | 158 | HSP90AB1 | 244 | PDPK1 | 330 | TNK1 |
| 73 | CPT2 | 159 | HTR1A | 245 | PIK3CA | 331 | TOP1 |
| 74 | CSNK2B | 160 | HTR2A | 246 | PIK3CB | 332 | TOP2A |
| 75 | CTSB | 161 | HTR2C | 247 | PIK3CD | 333 | TRIM24 |
| 76 | CTSD | 162 | HTR7 | 248 | PIK3CG | 334 | TRPA1 |
| 77 | CTSK | 163 | ICMT | 249 | PIK3R1 | 335 | TTR |
| 78 | CTSL | 164 | IDH1 | 250 | PIN1 | 336 | TYRO3 |
| 79 | CXCR4 | 165 | IDO1 | 251 | PLA2G2A | 337 | UGT2B7 |
| 80 | CYP2A6 | 166 | IMPDH2 | 252 | PLAT | 338 | ULK3 |
| 81 | CYP3A4 | 167 | ITGB1 | 253 | PLG | 339 | USP7 |
| 82 | CYSLTR2 | 168 | ITGB3 | 254 | PPARA | 340 | VDR |
| 83 | DCTPP1 | 169 | ITK | 255 | PRCP | 341 | WDR5 |
| 84 | DCUN1D1 | 170 | KAT6A | 256 | PRKACA | 342 | XDH |
| 85 | DHFR | 171 | KCNA5 | 257 | PRKCA | 343 | YES1 |
| 86 | DHODH | 172 | KCNH2 | 258 | PRKCB | 344 | ZAP70 |

**Table S16：Binding Energies of 12 Key Compounds with 15 Key Targets. The binding energy less than -1.2 kcal/mol is considered to have good binding ability.**

| **Mol ID** | **Key Components** | **Binding energy (kcal/mol)** | | | | | | | | | | | | | | |
| --- | --- | --- | --- | --- | --- | --- | --- | --- | --- | --- | --- | --- | --- | --- | --- | --- |
|  |  | **CXCR4** | **ESR1** | **GSK3B** | **HIF1A** | **HSP90AA1** | **HSP90AB1** | **KDR** | **MAPK3** | **MMP9** | **NFKB1** | **PTGS2** | **STAT3** | **TLR4** | **EP300** | **MTOR** |
| MOL000049 | 3β-acetoxyatractylone | -7.6 | -7.3 | -7.1 | -6.4 | -9.1 | -8.1 | -7.6 | -7.4 | -7.0 | -5.8 | -7.8 | -6.3 | -7.6 | -7.4 | -8.2 |
| MOL000072 | 8β-ethoxy atractylenolide | -7.1 | -8.7 | -7.2 | -7.0 | -7.8 | -7.5 | -6.7 | -7.4 | -7.6 | -6.2 | -7.7 | -6.5 | -8.2 | -8.1 | -7.2 |
| MOL000831 | Alisol B monoacetate | -8.2 | -7.1 | -7.7 | -7.6 | -7.6 | -7.7 | -6.6 | -7.5 | -8.3 | -6.7 | -8.0 | -7.1 | -9.3 | -7.6 | -7.4 |
| MOL000856 | Alisol C monoacetate | -8.3 | -8.0 | -8.1 | -7.4 | -7.6 | -7.9 | -7.3 | -6.9 | -7.1 | -7.5 | -8.1 | -7.3 | -9.7 | -6.7 | -7.6 |
| MOL000358 | β-sitosterol | -9.6 | -8.6 | -8.3 | -8.7 | -9.8 | -8.9 | -8.4 | -9.3 | -8.8 | -7.9 | -10.0 | -8.3 | -10.6 | -8.3 | -8.9 |
| MOL000296 | hederagenin | -9.2 | -6.9 | -8.7 | -7.5 | -7.4 | -6.8 | -8.7 | -6.8 | -6.8 | -7.1 | -8.2 | -6.6 | -9.9 | -7.5 | -8.4 |
| MOL000422 | Kaempferol | -8.3 | -8.3 | -8.1 | -6.6 | -8.9 | -8.9 | -8.0 | -8.7 | -9.1 | -7.2 | -9.0 | -7.1 | -8.1 | -8.9 | -7.5 |
| MOL001494 | Mandenol | -6.3 | -5.5 | -5.4 | -4.9 | -5.8 | -6.4 | -5.6 | -5.2 | -6.2 | -4.4 | -4.9 | -5.1 | -6.5 | -5.5 | -5.4 |
| MOL002135 | Myricanone | -5.9 | -5.0 | -5.5 | -4.8 | -6.6 | -6.5 | -5.3 | -5.2 | -5.3 | -4.1 | -4.8 | -4.2 | -6.0 | -6.2 | -6.0 |
| MOL001918 | Paeoniflorgenone | -8.4 | -8.3 | -7.6 | -6.8 | -8.4 | -8.8 | -7.4 | -8.5 | -7.8 | -6.8 | -8.9 | -7.1 | -8.5 | -8.3 | -7.4 |
| MOL000449 | Stigmasterol | -8.5 | -7.8 | -9.1 | -7.9 | -8.9 | -8.6 | -7.9 | -7.5 | -7.7 | -6.5 | -7.4 | -7.6 | -9.7 | -6.9 | -8.7 |
| MOL002157 | Wallichilide | -7.8 | -6.4 | -7.2 | -7.0 | -7.1 | -7.2 | -7.7 | -7.3 | -8.2 | -6.1 | -7.3 | -6.7 | -8.6 | -7.2 | -7.6 |
